# Effect of proprioceptive neuromuscular facilitation on pain and joint mobility in knee osteoarthritis: a systematic review and meta-analysis of randomized controlled trials

**DOI:** 10.7717/peerj.20581

**Published:** 2026-01-16

**Authors:** Zihang Hu, Jie Dong, Yixian Zeng, Zijun He, Qingwei Wang, Qinglu Luo

**Affiliations:** 1 Department of Rehabilitation Medicine, The Tenth Affiliated Hospital, Southern Medical University (Dongguan People’s Hospital), Dongguan, Guangdong, China; 2 Department of Rehabilitation Medicine, Dongguan Songshan Lake Central Hospital, Dongguan, Guangdong, China; 3 School of Sports Medicine and Rehabilitation, Beijing Sport University, Beijing, China; 4 Dongguan Experimental Centre for Sports Rehabilitation Research, Dongguan, Guangdong, China; 5 Dongguan Key Specialty of Traditional Chinese Medicine (Rehabilitation Department), Dongguan, Guangdong, China; 6 Department of Rehabilitation Medicine, The Fifth Affiliated Hospital of Guangzhou Medical University, Guangzhou, Guangdong, China

**Keywords:** Sports health, PNF, Knee osteoarthritis, Rehabilitation

## Abstract

**Objective:**

To systematically assess the effect of proprioceptive neuromuscular facilitation (PNF), compared to control interventions or other rehabilitation techniques (RT), on pain intensity and knee active range of motion (AROM) in adults with knee osteoarthritis (KOA).

**Methodology:**

This systematic review and meta-analysis was reported in accordance with PRISMA guidelines. A systematic search was conducted in PubMed, Embase, Web of Science, CENTRAL, CNKI, Wanfang, and VIP for studies published from database inception to August 2025. Randomized controlled trials comparing PNF with control interventions or RT were identified in adults with KOA. Primary outcomes were change in pain and change in AROM from baseline to post-intervention. Data were pooled using a random effects model, with risk of bias assessed using the Revised Cochrane risk-of-bias tool (RoB-2) and the certainty of evidence rated using the Grading of Recommendations, Assessment, Development, and Evaluations (GRADE) approach.

**Results:**

Five studies (*n* = 201) comparing PNF versus control and four studies (*n* = 202) comparing PNF versus RT were included. Meta-analysis was conducted only for comparisons between PNF and control. In addition, PNF was qualitatively compared with RT, which included soft tissue mobilization (two randomized controlled trials (RCTs)), neuromuscular exercise (one RCT), and stretching (one RCT). Compared to control interventions, PNF demonstrated significantly greater benefits in pain reduction (standardized mean difference (SMD) = −1.14, *p* < 0.001) and knee AROM improvement (weighted mean difference (WMD) = 10.08, *p* < 0.001), albeit the reduction in pain (WMD = −1.66, *p* < 0.001, four RCTs) did not reach the minimal clinically important difference (MCID). When compared with RT, findings for change in pain were mixed across four RCTs (one favored RT, two showed no difference, one favored PNF); for knee AROM, one favored PNF, while the other showed no difference (no pooling due to methodological heterogeneity).

**Conclusions:**

PNF appears to yield greater improvements in pain reduction and joint mobility compared to control interventions among individuals with KOA. While its efficacy relative to RT remains inconclusive, PNF shows potential as an alternative or adjunct rehabilitation approach. Further high-quality RCTs are needed to determine the effects of PNF on pain reduction and joint mobility in KOA.

## Introduction

Knee osteoarthritis (KOA) is a progressive degenerative disorder characterized by activity-related knee pain and functional impairment. With a lifetime risk for knee replacement of up to 30% (Burn et al., 2019), KOA has emerged as a significant contributor to the global osteoarthritis burden (Cao et al., 2024). Demographic shifts, including population growth and aging, are projected to drive a 74.9% increase in osteoarthritis cases by 2050 compared to 2020 levels (GBD 2021 Osteoarthritis Collaborators, 2023). Current KOA management guidelines recommend exercise, education, weight reduction (if applicable), and specific pharmacotherapy (*e.g.*, NSAIDs) as first-line symptomatic treatments (Duong et al., 2023). However, prolonged use of pharmacological treatments, such as non-steroidal anti-inflammatory drugs and opioids, is associated with increased adverse event risks (Da Costa et al., 2021). Surgical intervention is typically reserved for end-stage KOA, characterized by the absence of joint space or severe, unremitting pain, when conservative therapies fail to provide adequate symptom relief, leaving a substantial therapeutic gap (Duong et al., 2023; Srivastava, Surgical Management of Osteoarthritis of the Knee Work Group & Staff of the American Academy of Orthopaedic Surgeons, 2023). Given these limitations and the high disability-adjusted life years (DALYs) burden imposed by KOA (Li et al., 2024), there is an urgent need to explore targeted and effective non-pharmacological interventions to enhance patients’ quality of life and reduce societal burden.

Among non-pharmacological approaches, physical exercise is strongly recommended by multiple clinical guidelines for its efficacy in improving pain and functional outcomes (Katz, Arant & Loeser, 2021). The benefits of exercise encompass muscle strengthening, modulation of inflammatory and cartilage metabolism, pain reduction, and psychological state improvement. Proprioceptive neuromuscular facilitation (PNF), a specialized rehabilitation technique within the realm of physical exercise, employs specific movement patterns and manual stimulation to enhance muscle strength and proprioception, thereby improving motor flexibility and coordination (Engle & Canner, 1989). In KOA patients, PNF protocols typically emphasize resistance and relaxation techniques, integrating movement with lower limb patterns to optimize periarticular muscle strength and flexibility (Alaca, Atalay & Guven, 2015; Song et al., 2020; Gao et al., 2023).

Despite PNF’s demonstrated potential across various rehabilitation contexts (Liu et al., 2021; Zaworski & Latosiewicz, 2021; Hortobágyi et al., 2022), its efficacy in adult KOA patients remains insufficiently investigated. A recent network meta-analysis by Zhu, Chen & Belcastro (2024), comparing PNF with control interventions for pain management, suggested PNF’s effectiveness in pain reduction. However, this analysis was limited by the inclusion of only two randomized controlled trials (RCTs). Moreover, there is a paucity of systematic reviews comparing PNF with other rehabilitation techniques (RT). To address this gap, our systematic review and meta-analysis of RCTs aimed to evaluate the effects of PNF on pain intensity and knee active range of motion (AROM) in adult KOA patients. We prespecified two primary comparisons: PNF *versus* control interventions (usual/routine care with no structured, progressive exercise) and PNF *versus* RT. This systematic review and meta-analysis aimed to synthesize the current evidence on the efficacy of PNF in KOA management, potentially informing future research directions and clinical decision-making in KOA rehabilitation strategies.

## Methods

This meta-analysis was reported in accordance with the Preferred Reporting Items for Systematic Reviews and Meta-Analyses (PRISMA) guidelines (Page et al., 2021). The review protocol was registered in PROSPERO (CRD42024556418).

### Data sources and search strategies

To identify relevant RCTs, a systematic search was performed in the electronic databases Pubmed/Medline (NLM), Embase, Web of Science, CENTRAL, China National Knowledge Infrastructure (CNKI), Wanfang and VIP (from inception to April 2024), without language restriction. Our search was updated on August 15, 2025. The search was independently conducted by two investigators (ZH and YZ), who were responsible for saving the retrieved articles, removing duplicates, and screening titles, abstracts, and full texts. Any disagreements were resolved by consulting a third author (QL). The full search strategy, primarily focused on PNF and knee osteoarthritis, is documented in Table S1.

### Eligibility and exclusion criteria

Parallel RCTs were included if they met the inclusion criteria for this meta-analysis, which required PNF interventions to include at least one specific PNF technique (*e.g.*, rhythmic initiation, rhythmic stabilization, dynamic/stabilizing reversals, combination of isotonics, hold–relax, contract–relax, and hold–relax–agonist contraction/reciprocal inhibition). Use of spiral–diagonal movement patterns was permitted but not required. PNF intervention parameters had to report at least one of the following: duration, session length, or frequency. Our systematic review included two comparisons: PNF *vs.* control interventions and PNF *vs.* RT. The control group had to involve minimal or no physical activity for comparability with non-exercise conditions. Control interventions were defined as usual/routine care with no added structured, supervised, progressive exercise (*e.g.*, health counseling, no intervention, or passive activities) beyond shared baseline interventions. RT refers to comparators that added a structured, supervised, progressive rehabilitation program such as stretching, strength training, and exercise training, most of which are recommended by existing guidelines for KOA management (Kolasinski et al., 2020; Van Doormaal et al., 2020; Brophy & Fillingham, 2022; Zhu et al., 2023). Baseline intervention (when present) was common to both arms, not prespecified by us, and did not determine comparator classification. After excluding the baseline intervention, comparisons must adhere to the inclusion criteria for intervention and comparator as specified above. Studies with three or more arms were included if two of the arms met the inclusion criteria for intervention and comparator. The primary outcomes included changes in pain score and knee AROM from pre- to post-intervention, with pain scores obtained through relevant pain assessment scales, such as the Visual Analog Scale (VAS) or the Numeric Pain Rating Scale (NPRS).

The exclusion criteria included studies that lacked original data (*e.g.*, reviews, editorials, comments), studies where PNF interventions did not target the knee joint, and studies where, after removing the baseline intervention, the PNF or RT groups used multiple interventions instead of a single one (*e.g.*, the RT group used both quadricep exercise and passive stretching). Studies involving participants with knee osteoarthritis following total knee arthroplasty were also excluded due to concerns of population heterogeneity.

### Data extraction

All studies that met the inclusion criteria were subsequently reviewed by two independent investigators (ZH and YZ). The data extraction focused on the following key variables: author, publication year, country, intervention characteristics (duration, session length, frequency), reported outcomes (baseline and post-intervention means and SDs for pain score and knee AROM), and participant demographics (*e.g.*, age, gender, Kellgren-Lawrence (K/L) grade). The K/L grading system classifies knee osteoarthritis based on radiographic findings (Kellgren & Lawrence, 1957). Quantitative data were extracted in the form of mean ±   standard deviation. Missing outcome data were sought by contacting the corresponding authors *via* email. Where a study reported a single overall knee pain score (no laterality), we extracted that global, person-level value; where left and right knees were reported separately, we prespecified extracting the more symptomatic (higher-pain) knee as the index outcome. This approach aligns with common knee-OA practice—either analyze the worse (‘index’) knee or use questionnaires that do not distinguish whether pain is unilateral or bilateral—both intended to reflect the participant’s symptomatic burden (White et al., 2010; Kim et al., 2018). Any discrepancies during data extraction were resolved by a third investigator (JD). If the data were available only as graphs and lacked explicit means and standard deviations, they were extracted using WebPlotDigitizer Version 4.7 (Drevon, Fursa & Malcolm, 2017).

### Quality assessment

Two independent authors analysed the risk of bias (ZH and YZ), and a third (JD) was consulted if there were any discrepancies in the risk of bias assessments. The Revised Cochrane Risk-of-Bias tool for individually randomized, parallel-group trials (RoB 2) was used to assess the included studies (Sterne et al., 2019). The assessment focused on five key domains: the randomization process, deviations from intended interventions, missing outcome data, outcome measurement, and selection of the reported result. The overall risk of bias for each study was determined using the RoB 2 algorithm, and classified as “low risk,” “high risk,” or “some concerns”. Studies rated as low risk in all domains were classified as ‘low risk of bias.’ If at least one domain was rated as high risk, the study was classified as ‘high risk of bias.’ Studies with some concerns in one or more domains but without high risk were classified as ‘some concerns.’ Certainty of evidence was assessed by ZH using the Grading of Recommendations, Assessment, Development, and Evaluations (GRADE) approach for the PNF *vs.* control comparison (pain, knee AROM). RoB 2 assessments were performed for all included RCTs (PNF *vs.* control and PNF *vs.* RT).

### Statistical analysis

Standardized mean difference (SMD) or weighted mean difference (WMD) along with 95% confidence intervals (CIs) were calculated to compare the effects of PNF *vs.* control on pain scores and knee AROM. A random-effects model (DerSimonian and Laird method, DL) was used to pool effect sizes, and Hedge’s g was used to estimate effect size magnitude, with results displayed using forest plots. The within-group change scores were calculated following the Cochrane Handbook for Systematic Reviews of Interventions (Higgins et al., 2021). A correlation coefficient (Pearson’s *r*-value) of 0.5 between pre- and post-intervention data was assumed when calculating within-group SD (Follmann et al., 1992). Based on previous research (Kim et al., 2021; Salehi et al., 2023), the MCID for the VAS in our included participants was set at 2.5. Statistical heterogeneity was assessed using the I^2^ and tau^2^ statistics. Due to methodological heterogeneity, the results of PNF *vs.* RT were not included in the quantitative analysis. No subgroup analysis, meta-regression and assessment of publication bias were conducted because there were not enough included original studies available in the meta-analysis.

Trial Sequential Analysis (TSA) was performed to control for Type I and Type II errors. The following parameters were pre-specified: alpha level of 5%, beta of 10% (power of 90%), and the DL model. The mean difference and variance were based on the pooled observed standard deviation of the current meta-analysis. A *Z*-curve that does not cross boundaries and falls short of the required information size suggests the need for more research.

Sensitivity analysis was performed when the number of studies included in the comparison was ≥3, by sequentially removing each study to assess its impact on the overall results. Additionally, the robustness of findings was evaluated by varying the correlation coefficient (*r* = 0.3, *r* = 0.7) (Yamaoka & Tango, 2012). For cases with five or more included studies, Bayesian analysis was conducted as an additional assessment of robustness. The Bayesian model utilized the Markov Chain Monte Carlo (MCMC) method to generate posterior distributions, and a random-effects model was applied to estimate the posterior means (Hedge’s g) and 95% credible intervals (CrIs).

Quantitative analysis was conducted using a frequentist model in Stata 16.0 and a Bayesian model in R (Version 4.4.1). Additionally, TSA was performed for each outcome using a random-effects model with Trial Sequential Analysis v.0.9.5.10 beta software. A *p*-value of <0.05 was considered statistically significant.

## Results

**Figure 1:**
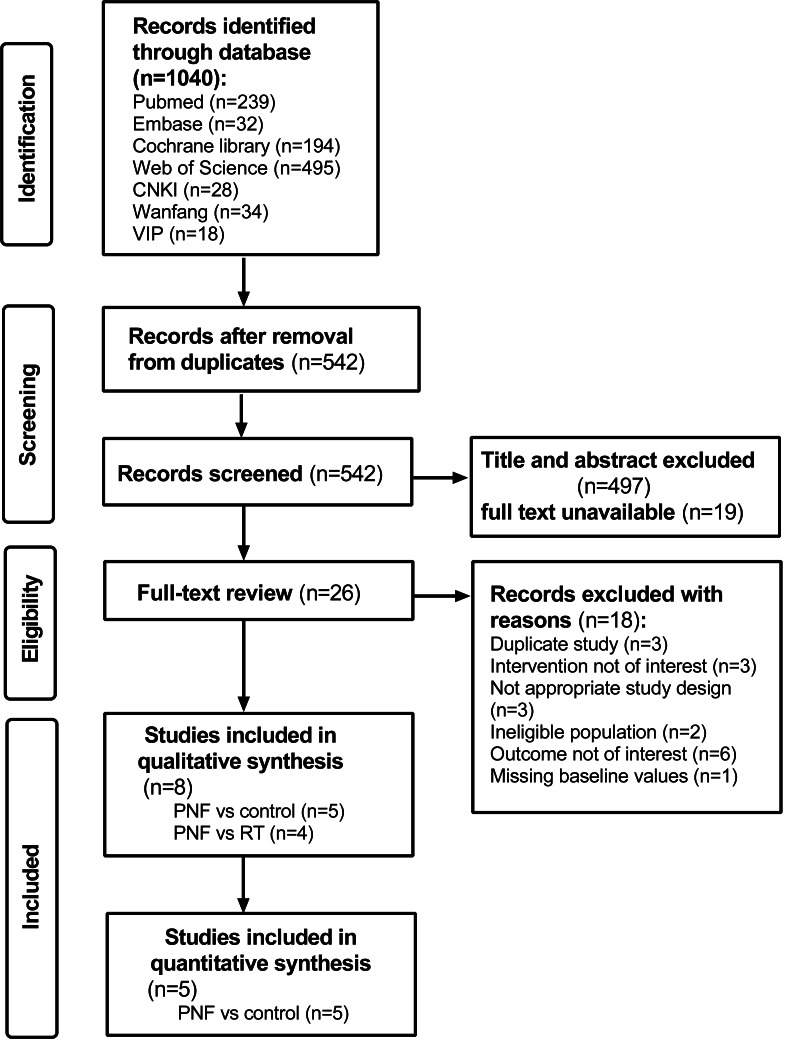
PRISMA flow diagram for a systematic review and meta-analysis of randomized clinical trials of PNF in knee osteoarthritis.

A total of 1,040 studies were identified through the literature search. After removing duplicates, 542 records were screened. Following a full-text assessment of 26 original studies, eight studies (Weng et al., 2009; Li et al., 2018; Song et al., 2020; Anjum et al., 2023; Gao et al., 2023; Nafees et al., 2023; Zhang et al., 2023; Shen et al., 2024) were included in the final analysis. Of these, five studies (Weng et al., 2009; Li et al., 2018; Song et al., 2020; Gao et al., 2023; Shen et al., 2024) compared PNF with a control group, and four studies (Weng et al., 2009; Anjum et al., 2023; Nafees et al., 2023a; Zhang et al., 2023) compared PNF with rehabilitation techniques (RT), with one study overlapping both comparisons. The studies comparing PNF with the control group (Weng et al., 2009; Li et al., 2018; Song et al., 2020; Gao et al., 2023; Shen et al., 2024) were included in the quantitative analysis. The search and inclusion process is presented in Fig. 1. The consistency between the two reviewers in data screening and integration was high (kappa = 0.899, 95% CI [0.759–1.039]), with values above 0.8 suggesting almost perfect agreement (Yamato et al., 2017).

### Study characteristics

The included studies’ characteristics are summarized in Table 1 and Table S2, providing an overview of participant demographics, intervention protocols, and outcome measures. The studies included in this review were published between 2009 and 2025. All studies comparing PNF with the control group were conducted in China (Weng et al., 2009; Li et al., 2018; Song et al., 2020; Gao et al., 2023; Shen et al., 2024). Of the four studies comparing PNF with RT (Weng et al., 2009; Anjum et al., 2023; Nafees et al., 2023; Zhang et al., 2023), two were conducted in China (Weng et al., 2009; Zhang et al., 2023) and two in Pakistan (Anjum et al., 2023; Nafees et al., 2023).

The PNF *vs.* control comparison included 201 participants across five studies (Weng et al., 2009; Li et al., 2018; Song et al., 2020; Gao et al., 2023; Shen et al., 2024), with a weighted average age of 66.1 years (range: 64–68 years). Women made up 57.1% of participants. All studies (Weng et al., 2009; Li et al., 2018; Song et al., 2020; Gao et al., 2023; Shen et al., 2024) selected patients with K/L grades II (minimal OA) or III (moderate OA) as part of their inclusion criteria. Three studies (Song et al., 2020; Gao et al., 2023; Shen et al., 2024) intervened with both lower limb spiral-diagonal movement patterns and specialized PNF techniques. The remaining two studies (Weng et al., 2009; Li et al., 2018) utilized specialized PNF techniques exclusively for stretching. PNF interventions had a mean duration of 7.2 weeks, with each session averaging 44 min. Most studies (four) (Weng et al., 2009; Song et al., 2020; Gao et al., 2023; Shen et al., 2024) performed PNF three times per week, while one study (Li et al., 2018) used a frequency of five times per week.

A total of 202 participants from four studies (Weng et al., 2009; Anjum et al., 2023; Nafees et al., 2023; Zhang et al., 2023) were included in the comparison between PNF and RT. The participants’ weighted average age was 51.6 year, and over two-thirds (71.7%) were women. Among the studies comparing PNF and RT, two compared PNF to soft tissue mobilization (Anjum et al., 2023; Nafees et al., 2023), one to neuromuscular exercise (Zhang et al., 2023), one to passive static stretching (Weng et al., 2009). In most comparisons (three out of four) (Weng et al., 2009; Anjum et al., 2023; Nafees et al., 2023), PNF was applied solely for knee stretching using specialized PNF techniques, while in the other two comparisons (Nafees et al., 2023; Zhang et al., 2023), both limb spiral-diagonal movement patterns and specialized PNF techniques were used. The average intervention duration was 5.3 weeks, with a frequency of 2.7 times per week. Each PNF session lasted an average of 38.3 min. Because all trials reported outcomes immediately post-intervention, pooled estimates reflect short-term (end-of-treatment) effects.

**Table 1:** General characteristics of all included studies.

**Study**	**K/L Grade**	**Sample size (M/F)**			**Mean age (years)**			**PNF session**				**Outcomes**
		PNF	Comparator		PNF	Comparator		Duration (wk)	Time (min)	Frequency (/wk)		
**Trials comparing PNF and control interventions**												
Shen et al. (2024)	I, II, III	14 (10/4)	13 (9/4)		65.3 ± 4.6	66.6 ± 7.0		6	60	3		VAS, knee AROM
Gao et al. (2023)	II, III, IV	13 (8/5)	14 (8/6)		68.5 ± 2.1	67.9 ± 1.4		8	60	3		VAS
Song et al. (2020)	I, II, III	13 (5/8)	16 (6/10)		68.5 ± 4.3	67.4 ± 3.4		12	60	3		VAS-WOMAC
Li et al. (2018)	II, III, IV	30 (10/20)	30 (11/19)		63.5 ± 8.8	64.3 ± 9.2		2	30	5		VAS
Weng et al. (2009)	II, III^#^	30	28					8	10	3		VAS, knee AROM
**Trials comparing PNF and rehabilitation technique (RT)**												
Anjum et al. (2023)	I, II	27 (9/18)	30 (5/25)		44.3 ± 4.9	45.9 ± 4.6		6	30	3		VAS
Zhang et al. (2023)	I, II	20 (8/12)	20 (8/12)		56.6 ± 4.0	57.9 ± 4.1		6	60	2		VAS, knee AROM
Nafees et al. (2023)		24 (8/16)	24 (3/21)		56.8 ± 8.8	52.1 ± 7.1		4	30	3		VAS
Weng et al. (2009)	II, III^#^	33	27					8	10	3		VAS, knee AROM

**Notes.**

Abbreviations K/L Grade Kellgren–Lawrence grade PNF proprioceptive neuromuscular facilitation VAS visual analogue scale AROM active range of motion used when the knee flexes WOMAC Western Ontario and McMaster Universities Osteoarthritis Index NPRS numerical pain rating scale

Quantitative data are presented as mean ± SD.

# The K/L grade was derived from the equivalent Altman Grade II classification.

### Risk of bias with studies

Figure S1 presents a summary of the risk of bias within the included studies. For the five studies comparing PNF with the control group (Weng et al., 2009; Li et al., 2018; Song et al., 2020; Gao et al., 2023; Shen et al., 2024), three studies (Weng et al., 2009; Gao et al., 2023; Shen et al., 2024) reported sufficient details on the randomization process, and all studies (Weng et al., 2009; Li et al., 2018; Song et al., 2020; Gao et al., 2023; Shen et al., 2024) appropriately handled missing data. Two studies (Weng et al., 2009; Gao et al., 2023) used suitable methods to measure outcomes. However, all studies (Weng et al., 2009; Li et al., 2018; Song et al., 2020; Gao et al., 2023; Shen et al., 2024) had concerns for a risk of bias related to deviations from intended interventions. One study (Gao et al., 2023) had a high risk for selectively report outcomes. Overall, four studies (Weng et al., 2009; Li et al., 2018; Song et al., 2020; Shen et al., 2024) were rated as ‘some concerns’, while one (Gao et al., 2023) as ‘high risk.’ The main sources of bias were deviations from intended interventions and selective outcome reporting.

In the four studies comparing PNF with RT (Weng et al., 2009; Anjum et al., 2023; Nafees et al., 2023; Zhang et al., 2023), three studies (Weng et al., 2009; Anjum et al., 2023; Nafees et al., 2023) reported sufficient details on the randomization process. Three studies (Weng et al., 2009; Nafees et al., 2023; Zhang et al., 2023) appropriately handled missing data and four (Weng et al., 2009; Anjum et al., 2023; Zhang et al., 2023; Nafees et al., 2023) reported complete, non-selective outcome. However, four studies (Weng et al., 2009; Anjum et al., 2023; Nafees et al., 2023; Zhang et al., 2023) exhibited a certain degree of risk of bias due to deviation from the expected interventions, while two (Anjum et al., 2023; Nafees et al., 2023) raised concerns regarding the risk of bias in outcome measurement. Overall, one (Anjum et al., 2023) were classified as ‘low risk’, and the rest (Weng et al., 2009; Nafees et al., 2023; Zhang et al., 2023) raised ‘some concerns’. The primary source of bias was related to the deviations from intended interventions.

### Effects of PNF on pain score and knee AROM

#### Change in pain score

**Figure 2:**
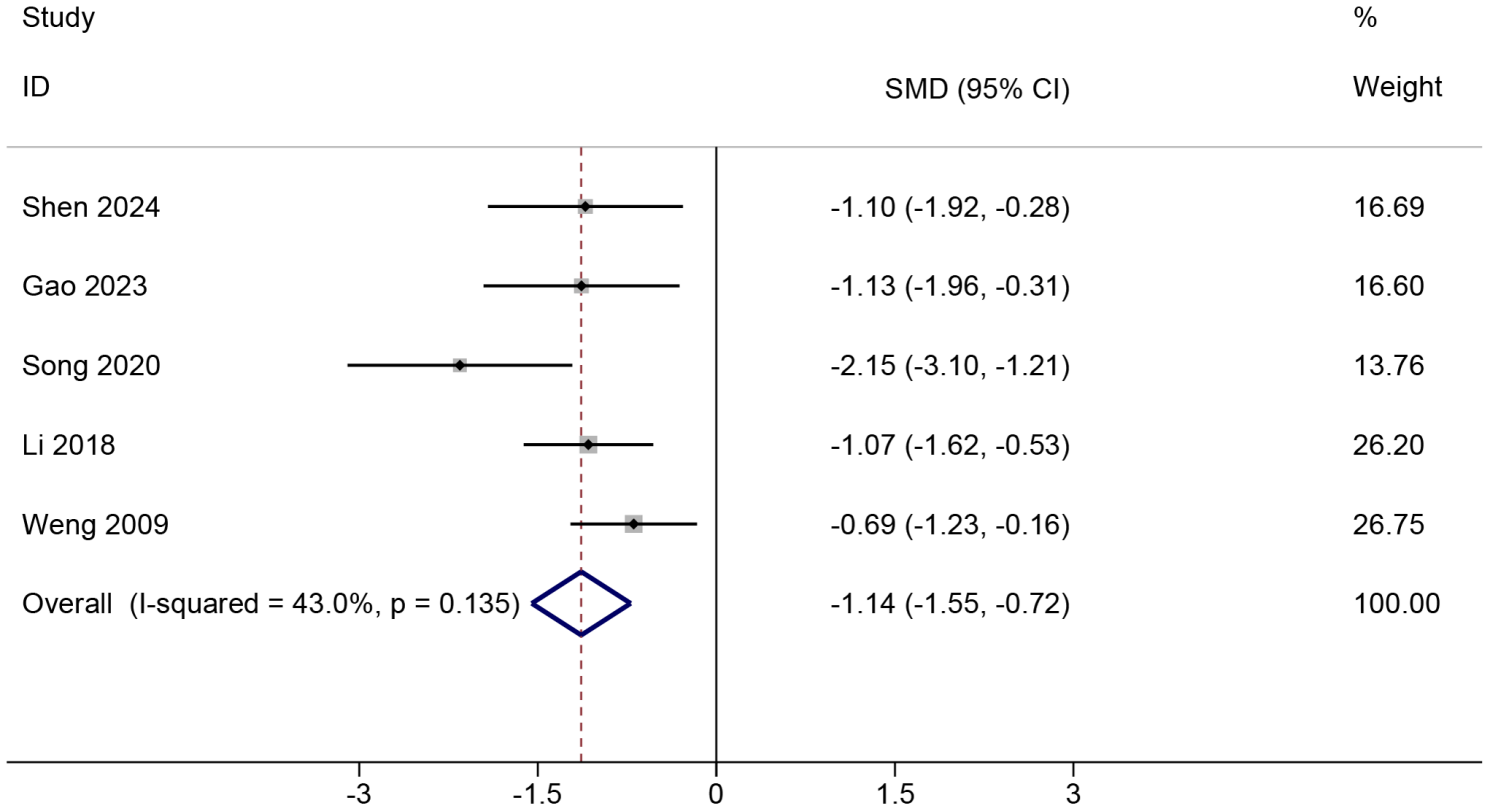
Comparison of pain score changes from baseline to post-intervention between the PNF and control groups. The random-effects model was based on the DerSimonian and Laird (DL) method. Black dots represent the mean pain score changes for each study, with horizontal lines indicating the 95% confidence intervals (CIs), and the size of the black dots reflecting the study weight. Diamonds represent the pooled estimates, with their outer points indicating the 95% CIs. Note: Weng et al. (2009); Li et al. (2018); Song et al. (2020); Gao et al. (2023); Shen et al. (2024).

All studies (Weng et al., 2009; Li et al., 2018; Song et al., 2020; Gao et al., 2023; Shen et al., 2024) comparing PNF with control reported pain score values and could be meta-analyzed, of which four studies (Weng et al., 2009; Li et al., 2018; Gao et al., 2023; Shen et al., 2024) reported VAS score, and the remaining (Song et al., 2020) presented VAS-WOMAC (Table S2). PNF demonstrated a significant improvement in change in pain score compared to the control condition (SMD = −1.14, 95%Cl [−1.55 to −0.72], *p* < 0.001; GRADE = very low; Fig. 2). The certainty of evidence was downgraded for risk of bias, indirectness, and imprecision (Table S3). Moderate heterogeneity was observed (Chi^2^ = 7.02, *p* = 0.135, tau^2^ = 0.09, I^2^=43%). In VAS-only trials (Weng et al., 2009; Li et al., 2018; Gao et al., 2023; Shen et al., 2024), the pooled effect size (WMD = −1.66, 95%Cl [−2.18 to −1.14], *p* < 0.001; 4 RCTs) did not reach the predefined MCID of 2.5, indicating that the improvement may not be clinically significant (Fig. S2). TSA was done in studies reporting on change in VAS, and showed that the required information size was attained (Fig. S3). The effect of PNF, as estimated by the Bayesian model (SMD = −1.16, 95% CrI [−1.86 to −0.53], tau^2^ = 0.39), was consistent with the results obtained using the frequentist approach. Sensitivity analysis indicated that the meta-analysis results were robust. Neither the exclusion of any single study nor the adjustment of the correlation coefficient (to either 0.3 or 0.7) altered the overall findings, demonstrating the stability of our results across various analytical scenarios (Figs. S4 and S5).

In studies (Weng et al., 2009; Anjum et al., 2023; Nafees et al., 2023; Zhang et al., 2023) comparing the effects of PNF *versus* RT on change scores for pain, four articles (Weng et al., 2009; Anjum et al., 2023; Nafees et al., 2023; Zhang et al., 2023) reported outcomes using the VAS (Table S2). Due to substantial methodological heterogeneity within the RT group, we did not directly pool the change score results for this comparison. Across four RCTs, the direction of effects for change in pain was mixed—one favored RT, two showed no difference, and one favored PNF (Table S4). PNF showed no superiority over passive RT interventions, such as soft tissue mobilization (Anjum et al., 2023; Nafees et al., 2023) and stretching (Weng et al., 2009) in reducing pain. Nafees et al. (2023) found no statistically significant difference in VAS change scores between PNF and dynamic soft tissue mobilization (Δ VAS mean ± SD: −5.31 ± 1.27 *vs.* −5.11 ± 1.36). Similarly, Weng et al. (2009) reported no significant difference between PNF and passive bilateral knee stretching in patients with severe knee joint symptoms (Δ VAS mean ± SD: −2.20 ± 1.71 *vs.* −1.60 ± 1.06). Additionally, Anjum et al. (2023) found that instrument soft tissue mobilization was more effective than PNF in reducing pain (Δ VAS mean±SD: −6.20 ± 1.10 *vs.* −3.61 ± 1.20). Although instrument soft tissue mobilization was more effective than PNF in some studies, the observed differences were small. Conversely, compared to neuromuscular training, PNF showed an advantage in alleviating pain associated with knee osteoarthritis (Δ VAS mean±SD: −3.19 ±  1.03 *vs.* −0.98 ± 1.21) (Zhang et al., 2023).

#### Change in knee AROM

**Figure 3:**
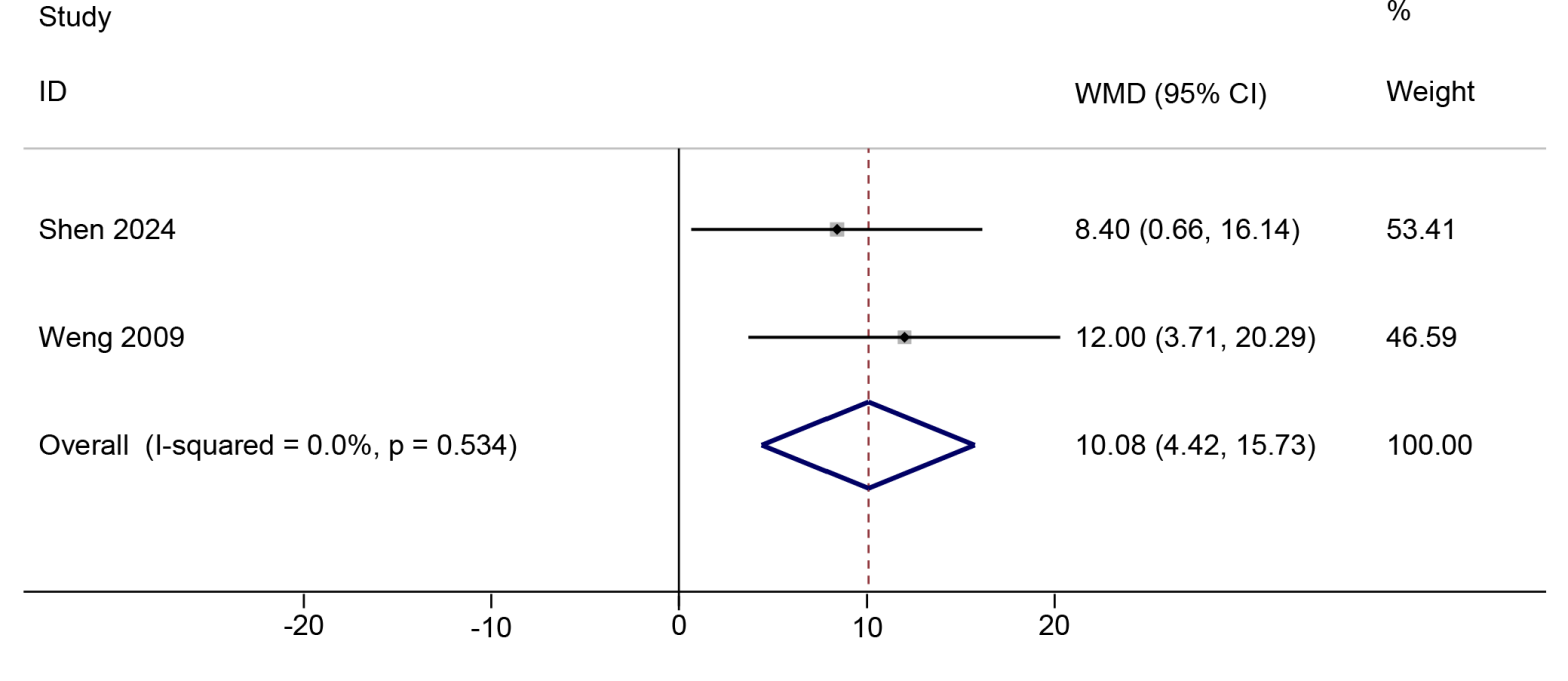
Comparison of knee AROM changes from baseline to post-intervention between the PNF and control groups. The DL random-effects model was used. Black dots indicate mean changes, horizontal lines show 95% CIs, and dot sizes reflect study weights. Diamonds denote pooled estimates with outer points indicating 95% CIs. Note: Weng et al. (2009); Shen et al. (2024).

Two studies (Weng et al., 2009; Shen et al., 2024) comparing PNF with the control condition reported change scores for knee AROM at immediate post-intervention (Table S2). PNF showed a positive effect compared to the control in improving change in knee AROM (WMD = 10.01, 95%Cl [4.42, 15.73], *p* < 0.001; GRADE = very low; Fig. 3). The certainty of evidence was downgraded for indirectness and serious imprecision (Table S3). No significant heterogeneity was observed (Chi^2^ = 0.39, *p* = 0.53, *τ*^2^ < 0.01, I^2^ < 0.1%). The TSA *Z*-curve crossed both the required information size (RIS) and conventional significance boundaries, indicating that the evidence is sufficient to support PNF’s positive effect on knee AROM (Fig. S6). Further sensitivity analysis was conducted by adjusting the correlation coefficient to 0.3 and 0.7. The results remained consistent across these adjustments, indicating the robustness of our findings (Fig. S7).

Two studies (Weng et al., 2009; Zhang et al., 2023) reported post-intervention knee AROM change values for PNF *versus* RT (Table S2). Similar to the pain score results, due to substantial methodological heterogeneity within the RT group, which employed various rehabilitation techniques, we did not pool the effect sizes for change in knee AROM. For change in knee AROM, a statistically significant difference favoring PNF was observed only in the comparison with neuromuscular training (Δ AROM mean±SD: 7.13 ± 5.69 *vs.* 3.26 ±  5.65) (Zhang et al., 2023). In contrast, no statistically significant difference was found between PNF and combined quadriceps and hamstring stretching (Δ AROM mean±SD: 17.0 ± 16.50 *vs.* 10.0 ± 14.40) (Weng et al., 2009) (Table S4).

## Discussion

In this meta-analysis, we evaluated the efficacy of PNF in change in pain and change in AROM in individuals with KOA at the immediate post-intervention time point. Compared to control interventions, PNF showed significant improvements in pain relief and increases in knee AROM immediately after treatment. In contrast, a narrative synthesis of PNF *versus* RT (*i.e.,* soft tissue mobilization, neuromuscular exercise and stretching) revealed inconclusive results for pain reduction and showed no consistent superiority of PNF in improving knee AROM.

### PNF *versus* control interventions

Our preliminary meta-analytic finding found evidence of both pain reduction and knee AROM improvements in KOA adults, strengthening case for PNF as an effective tool for pain management and joint mobility improvement in these populations. By GRADE, certainty for both outcomes at post-intervention was very low (Table S3), and should be interpreted as tentative. Consistent with our findings, a network meta-analysis including two studies confirmed the effectiveness of PNF in alleviating pain by the end of the treatment course, aligning with its superiority over controls (Zhu, Chen & Belcastro, 2024). Similarly, the direction of effect for PNF *versus* control aligns with the broader KOA literature, wherein exercise-based interventions generally outperform non-exercise comparators (Holden et al., 2023; Zhang et al., 2025). However, high-quality evidence regarding the long-term impact of PNF on physical function and quality of life in KOA patients remains scarce. The observed improvements in pain reduction and knee AROM is clinically relevant, as pain severity is strongly associated with patients’ functional status and quality of life (Nian et al., 2019). Pain worsening is also a key predictor of reduced physical function (De Rooij et al., 2016). Knee AROM may mediate the relationship between pain and long-term outcomes, as a cross-sectional study by Zifchock, Kirane & Hillstrom (2011). Revealed a significant negative correlation between knee pain levels and both range of motion and joint function. This suggests that pain exacerbation may lead to further restricted range of motion, thereby impacting patients’ daily activities and overall function.

### PNF *versus* RT

Results were inconclusive when comparing PNF with RT (*i.e.*, soft tissue mobilization, neuromuscular exercise and stretching) for pain relief and change in knee AROM. Nonetheless, these comparisons provide valuable insights to help clinicians and physiotherapists in making informed decisions with patients by discussing other treatment options along with PNF. Our findings indicate that when pain is considered as the primary intervention outcome, PNF shows no additional benefits over passive rehabilitation techniques recommended by current guidelines (Kolasinski et al., 2020; Van Doormaal et al., 2020; Brophy & Fillingham, 2022; Zhu et al., 2023). Notably, one study reported a potential advantage of PNF over active rehabilitation techniques (*i.e.*, neuromuscular training) in alleviating pain (Zhang et al., 2023). This may be attributed to the limited effects of active training on pain reduction, as pain improvement is not solely dependent on muscle strength gains (Bokaeian et al., 2018). For change in knee AROM, PNF and RT (*i.e.*, neuromuscular training (Zhang et al., 2023) and stretching (Weng et al., 2009)) showed similar gains, with the observed effect sizes being small to moderate and accompanied by wide confidence intervals (Table S4). This suggests that PNF may be included as one effective component of a rehabilitation plan for KOA; as such, it is not superior to the existing RT in improving knee AROM.

### Practical implications

Our findings indicate that a 6–12 week PNF intervention reduces post-intervention VAS scores by 1.66 points in KOA patients compared to controls (Fig. S2). Previous network meta-analyses have demonstrated that PNF showed a large effect compared to control interventions, with an SMD of 2.54 (Zhu, Chen & Belcastro, 2024), highlighting its substantial impact on KOA-associated pain. Given the lack of evidence supporting improvements in knee-related muscle strength, we speculate that PNF alleviates pain primarily by promoting the transformation of vastus lateralis IIB fibers into IIA fibers, facilitating adaptation toward more efficient and fatigue-resistant muscle properties (Kofotolis et al., 2005). The resisted end-range contractions used in PNF may transiently increase stretch tolerance *via* autogenic and reciprocal inhibition, and may modulate nociception through proprioceptive gating—mechanisms that could help explain short-term improvements in ROM and pain (Hindle et al., 2012). However, our pooled analysis revealed that the impact of PNF on VAS did not meet the predefined MCID value of 2.5, suggesting that current PNF protocols may not produce sufficient pain relief to significantly influence functional activities in KOA patients. Additionally, PNF was found to increase post-intervention AROM by 10.08° (Fig. 3), a magnitude comparable to the improvement in knee extension achieved with PNF in individuals with hamstring shortening (Youdas et al., 2010). This finding highlights the potential role of PNF in addressing muscle shortening and improving joint flexibility in KOA patients.

Our study also suggests that PNF may be considered one of several comparable options to RT, particularly when targeting knee AROM, since both interventions demonstrated comparable, rather than superior, effectiveness. Of note, when pain is the primary intervention target, practitioners should comprehensively evaluate the advantages and limitations of various rehabilitation techniques, with a focus on their effectiveness in alleviating pain, to determine whether to incorporate PNF into the treatment plan or combine it with other approaches for better outcomes. The superiority of PNF over neuromuscular training in pain relief was observed only in the RCT by Zhang et al. (2023) that lacked a baseline intervention, while other studies (Weng et al., 2009; Anjum et al., 2023; Nafees et al., 2023) comparing PNF and RT did not demonstrate such superiority. This may be attributed to interaction effects between the baseline intervention and the interventions being compared. Furthermore, incorporating PNF into existing rehabilitation protocols may offer additional benefits. For instance, Gökşen et al. (2021)’s study demonstrated that adding combined isotonic contraction to conventional physical therapy enhanced quadriceps strength and improved knee proprioception, compared to conventional techniques alone. However, these findings should be interpreted cautiously given the very low certainty and small samples. Overall, PNF may be considered one of several exercise options, rather than a preferred treatment over other prescribed exercises.

### Strengths, limitations and future research

To the best of our knowledge, this is the first meta-analysis to assess the short-term efficacy of PNF (*versus* control) in improving pain and AROM in KOA patients, as well as the first systematic review to evaluate the effects of PNF *versus* RT on pain relief and knee AROM in this population. The use of ROB-2 to assess the risk of bias, the inclusion of studies without restrictions on publication language, and the robustness of the findings serve as significant strengths of the present work. Based on existing evidence, our review offers practical clinical insights into the application of PNF for KOA rehabilitation.

Even so, this review has specific limitations. First, sample sizes were often small, with 55% of trials involving 50 or fewer participants, and the majority of studies focused on Asian populations, particularly Chinese participants in the PNF *vs.* control comparisons, which limits the generalizability of the findings. Second, while the TSA results for PNF *vs.* control on VAS indicated a statistically significant effect of PNF on pain reduction, most of the included studies showed potential concerns for risk of bias, necessitating additional high-quality studies to confirm this effect. Third, all trials only measured outcomes immediately after the intervention, without considering the duration of the effects. Thus, larger, multicenter trials with rigorous designs and extended follow-up periods are needed to validate the long-term pain-relieving effects of PNF in KOA patients. Fourth, variations in intervention protocols (*e.g.*, components, timing, duration, and frequency) and in control conditions likely contributed to heterogeneity. For instance, some studies (Weng et al., 2009; Li et al., 2018; Anjum et al., 2023; Nafees et al., 2023) focused on PNF techniques such as contract-relax and hold-relax, without incorporating more comprehensive techniques like rhythmic stabilization or dynamic reversal. Although specific PNF techniques varied, these differences reflect variations within the same intervention construct (PNF). We addressed this using a random-effects model and sensitivity analyses (leave-one-out; varying pre–post correlation), with a Bayesian random-effects check yielding similar inferences. Finally, the absence of subgroup analysis based on baseline interventions, due to insufficient studies, may have masked differences between PNF and controls. Interaction effects could exist between target and baseline interventions, or a ceiling effect from the baseline intervention may have limited the measurable impact of the target intervention. Future research should address these limitations by increasing the quantity and quality of studies, enabling more detailed and robust analyses to better understand the effects of PNF in diverse contexts.

## Conclusion

The present study suggests that PNF shows short-term efficacy over control interventions for pain reduction in individuals with KOA. By GRADE, the certainty of evidence for both pain and knee AROM is very low, so these estimates should be interpreted as tentative and confirmed in larger, rigorously designed RCTs with follow-up. Additionally, several rehabilitation techniques may be considered alternatives to PNF for improving knee mobility, with inconclusive differences for pain. Future studies should delineate the optimal components, timing, and duration of PNF and clarify its comparative advantages over conventional rehabilitation techniques.

##  Supplemental Information

10.7717/peerj.20581/supp-1 Supplemental Information 1 Supplementary materials

10.7717/peerj.20581/supp-2 Supplemental Information 2 Code: R

10.7717/peerj.20581/supp-3 Supplemental Information 3 Code:STATA

10.7717/peerj.20581/supp-4 Supplemental Information 4 PRISMA checklist

10.7717/peerj.20581/supp-5 Supplemental Information 5 Data

10.7717/peerj.20581/supp-6 Supplemental Information 6 Intended audience

## Additional Information and Declarations

### Data Availability

The following information was supplied regarding data availability: The raw data and codes are available in the Supplemental Files. This is a Systematic review/Meta-analysis.

## References

[ref-1] Alaca N, Atalay A, Guven Z (2015). Comparison of the long-term effectiveness of progressive neuromuscular facilitation and continuous passive motion therapies after total knee arthroplasty. Journal of Physical Therapy Science.

[ref-2] Anjum N, Sheikh RK, Omer A, Anwar K, Khan MMH, Aftab A, Awan WA (2023). Comparison of instrument-assisted soft tissue mobilization and proprioceptive neuromuscular stretching on hamstring flexibility in patients with knee osteoarthritis. PeerJ.

[ref-3] Bokaeian HR, Bakhtiary AH, Mirmohammadkhani M, Moghimi J (2018). Quadriceps strengthening exercises may not change pain and function in knee osteoarthritis. Journal of Bodywork and Movement Therapies.

[ref-4] Brophy RH, Fillingham YA (2022). AAOS clinical practice guideline summary: management of osteoarthritis of the knee (nonarthroplasty), third edition. The Journal of the American Academy of Orthopaedic Surgeons.

[ref-5] Burn E, Murray DW, Hawker GA, Pinedo-Villanueva R, Prieto-Alhambra D (2019). Lifetime risk of knee and hip replacement following a GP diagnosis of osteoarthritis: a real-world cohort study. Osteoarthritis and Cartilage.

[ref-6] Cao F, Xu Z, Li X-X, Fu Z-Y, Han R-Y, Zhang J-L, Wang P, Hou S, Pan H-F (2024). Trends and cross-country inequalities in the global burden of osteoarthritis, 1990–2019: a population-based study. Ageing Research Reviews.

[ref-7] Da Costa BR, Pereira TV, Saadat P, Rudnicki M, Iskander SM, Bodmer NS, Bobos P, Gao L, Kiyomoto HD, Montezuma T, Almeida MO, Cheng P-S, Hincapié CA, Hari R, Sutton AJ, Tugwell P, Hawker GA, Jüni P (2021). Effectiveness and safety of non-steroidal anti-inflammatory drugs and opioid treatment for knee and hip osteoarthritis: network meta-analysis. BMJ.

[ref-8] De Rooij M, vander Leeden M, Heymans MW, Holla JFM, Häkkinen A, Lems WF, Roorda LD, Veenhof C, Sanchez-Ramirez DC, De Vet HCW, Dekker J (2016). Prognosis of pain and physical functioning in patients with knee osteoarthritis: a systematic review and meta-analysis. Arthritis Care & Research.

[ref-9] Drevon D, Fursa SR, Malcolm AL (2017). Intercoder reliability and validity of webplotdigitizer in extracting graphed data. Behavior Modification.

[ref-10] Duong V, Oo WM, Ding C, Culvenor AG, Hunter DJ (2023). Evaluation and treatment of knee pain: a review. JAMA.

[ref-11] Engle RP, Canner GC (1989). Proprioceptive Neuromuscular Facilitation (PNF) and modified procedures for Anterior Cruciate Ligament (ACL) instability. The Journal of Orthopaedic and Sports Physical Therapy.

[ref-12] Follmann D, Elliott P, Suh I, Cutler J (1992). Variance imputation for overviews of clinical trials with continuous response. Journal of Clinical Epidemiology.

[ref-13] Gao B, Li L, Shen P, Zhou Z, Xu P, Sun W, Zhang C, Song Q (2023). Effects of proprioceptive neuromuscular facilitation stretching in relieving pain and balancing knee loading during stepping over obstacles among older adults with knee osteoarthritis: a randomized controlled trial. PLOS ONE.

[ref-14] GBD 2021 Osteoarthritis Collaborators (2023). Global, regional, and national burden of osteoarthritis, 1990–2020 and projections to 2050: a systematic analysis for the Global Burden of Disease Study 2021. The Lancet. Rheumatology.

[ref-15] Gökşen A, Can F, Yılmaz S, Korkusuz F (2021). Comparison of different neuromuscular facilitation techniques and conventional physiotherapy in knee osteoarthritis. Turkish Journal of Medical Sciences.

[ref-16] Higgins J, Thomas J, Chandler J, Cumpston M, Li T, Page M, Welch V (2021). Section 6.5.2.8, combining data from repeated measures. Cochrane handbook for systematic reviews of interventions version 6.2.

[ref-17] Hindle KB, Whitcomb TJ, Briggs WO, Hong J (2012). Proprioceptive Neuromuscular Facilitation (PNF): its mechanisms and effects on range of motion and muscular function. Journal of Human Kinetics.

[ref-18] Holden MA, Hattle M, Runhaar J, Riley RD, Healey EL, Quicke J, van der Windt DA, Dziedzic K, van Middelkoop M, Burke D, Corp N, Legha A, Bierma-Zeinstra S, Foster NE (2023). Moderators of the effect of therapeutic exercise for knee and hip osteoarthritis: a systematic review and individual participant data meta-analysis. The Lancet. Rheumatology.

[ref-19] Hortobágyi T, Ács P, Baumann P, Borbély G, Áfra G, Reichardt-Varga E, Sántha G, Tollár J (2022). Comparative effectiveness of 4 exercise interventions followed by 2 years of exercise maintenance in multiple sclerosis: a randomized controlled trial. Archives of Physical Medicine and Rehabilitation.

[ref-20] Katz JN, Arant KR, Loeser RF (2021). Diagnosis and treatment of hip and knee osteoarthritis: a review. JAMA.

[ref-21] Kellgren JH, Lawrence JS (1957). Radiological assessment of osteo-arthrosis. Annals of the Rheumatic Diseases.

[ref-22] Kim D, Park G, Kuo L-T, Park W (2018). The effects of pain on quadriceps strength, joint proprioception and dynamic balance among women aged 65 to 75 years with knee osteoarthritis. BMC Geriatrics.

[ref-23] Kim MS, Koh IJ, Choi KY, Seo JY, In Y (2021). Minimal clinically important differences for patient-reported outcomes after TKA depend on central sensitization. The Journal of Bone and Joint Surgery. American Volume.

[ref-24] Kofotolis N, Vrabas IS, Vamvakoudis E, Papanikolaou A, Mandroukas K (2005). Proprioceptive neuromuscular facilitation training induced alterations in muscle fibre type and cross sectional area. British Journal of Sports Medicine.

[ref-25] Kolasinski SL, Neogi T, Hochberg MC, Oatis C, Guyatt G, Block J, Callahan L, Copenhaver C, Dodge C, Felson D, Gellar K, Harvey WF, Hawker G, Herzig E, Kwoh CK, Nelson AE, Samuels J, Scanzello C, White D, Wise B, Altman RD, Di Renzo D, Fontanarosa J, Giradi G, Ishimori M, Misra D, Shah AA, Shmagel AK, Thoma LM, Turgunbaev M, Turner AS, Reston J (2020). 2019 American College of Rheumatology/Arthritis Foundation Guideline for the management of osteoarthritis of the hand, hip, and knee. Arthritis & Rheumatology.

[ref-26] Li E, Tan J, Xu K, Pan Y, Xu P (2024). Global burden and socioeconomic impact of knee osteoarthritis: a comprehensive analysis. Frontiers in Medicine.

[ref-27] Li Z, Yu J, Zhao C, Huang L (2018). Proprioceptive neuromuscular facilitation combined with interferential therapy and magnetic-fields-vibration heating system in patients with knee osteoarthritis. Military Medical Journal of South China.

[ref-28] Liu K, Yu X, Cui X, Su Y, Sun L, Yang J, Han W (2021). Effects of proprioceptive neuromuscular facilitation stretching combined with aerobic training on pulmonary function in copd patients: a randomized controlled trial. International Journal of Chronic Obstructive Pulmonary Disease.

[ref-29] Nafees K, Baig AAM, Ali SS, Ishaque F (2023). Dynamic soft tissue mobilization versus proprioceptive neuromuscular facilitation in reducing hamstring muscle tightness in patients with knee osteoarthritis: a randomized control trial. BMC Musculoskeletal Disorders.

[ref-30] Nian X, He Y, Ji Y, Huang Y, Sun E, Li L (2019). Associations between pain patterns and self-reported clinical outcomes in patients with knee osteoarthritis. Pain Medicine.

[ref-31] Page MJ, McKenzie JE, Bossuyt PM, Boutron I, Hoffmann TC, Mulrow CD, Shamseer L, Tetzlaff JM, Akl EA, Brennan SE, Chou R, Glanville J, Grimshaw JM, Hróbjartsson A, Lalu MM, Li T, Loder EW, Mayo-Wilson E, McDonald S, McGuinness LA, Stewart LA, Thomas J, Tricco AC, Welch VA, Whiting P, Moher D (2021). The PRISMA 2020 statement: an updated guideline for reporting systematic reviews. BMJ.

[ref-32] Salehi R, Valizadeh L, Negahban H, Karimi M, Goharpey S, Shahali S (2023). The Western Ontario and McMaster Universities Osteoarthritis, Lequesne Algofunctional index, arthritis impact measurement scale-short form, and visual analogue scale in patients with knee osteoarthritis: responsiveness and minimal clinically important differences. Disability and Rehabilitation.

[ref-33] Shen P, Zhao S, Xiao Y, Luo X, Mao D, Song Q (2024). Rehabilitation effects of PNF training on elderly patients with knee osteoarthritis. Journal of Shandong Institute of Physical Education and Sports.

[ref-34] Song Q, Shen P, Mao M, Sun W, Zhang C, Li L (2020). Proprioceptive neuromuscular facilitation improves pain and descending mechanics among elderly with knee osteoarthritis. Scandinavian Journal of Medicine & Science in Sports.

[ref-35] Srivastava AK, Surgical Management of Osteoarthritis of the Knee Work Group, Staff of the American Academy of Orthopaedic Surgeons (2023). American academy of orthopaedic surgeons clinical practice guideline summary of surgical management of osteoarthritis of the knee. The Journal of the American Academy of Orthopaedic Surgeons.

[ref-36] Sterne JAC, Savović J, Page MJ, Elbers RG, Blencowe NS, Boutron I, Cates CJ, Cheng H-Y, Corbett MS, Eldridge SM, Emberson JR, Hernán MA, Hopewell S, Hróbjartsson A, Junqueira DR, Jüni P, Kirkham JJ, Lasserson T, Li T, McAleenan A, Reeves BC, Shepperd S, Shrier I, Stewart LA, Tilling K, White IR, Whiting PF, Higgins JPT (2019). RoB 2: a revised tool for assessing risk of bias in randomised trials. BMJ.

[ref-37] Van Doormaal MCM, Meerhoff GA, Vliet Vlieland TPM, Peter WF (2020). A clinical practice guideline for physical therapy in patients with hip or knee osteoarthritis. Musculoskeletal Care.

[ref-38] Weng M-C, Lee C-L, Chen C-H, Hsu J-J, Lee W-D, Huang M-H, Chen T-W (2009). Effects of different stretching techniques on the outcomes of isokinetic exercise in patients with knee osteoarthritis. The Kaohsiung Journal of Medical Sciences.

[ref-39] White DK, Zhang Y, Felson DT, Niu J, Keysor JJ, Nevitt MC, Lewis CE, Torner JC, Neogi T (2010). The independent effect of pain in one versus two knees on the presence of low physical function in a multicenter knee osteoarthritis study. Arthritis Care & Research.

[ref-40] Yamaoka K, Tango T (2012). Effects of lifestyle modification on metabolic syndrome: a systematic review and meta-analysis. BMC Medicine.

[ref-41] Yamato TP, Maher C, Koes B, Moseley A (2017). The PEDro scale had acceptably high convergent validity, construct validity, and interrater reliability in evaluating methodological quality of pharmaceutical trials. Journal of Clinical Epidemiology.

[ref-42] Youdas JW, Haeflinger KM, Kreun MK, Holloway AM, Kramer CM, Hollman JH (2010). The efficacy of two modified proprioceptive neuromuscular facilitation stretching techniques in subjects with reduced hamstring muscle length. Physiotherapy Theory and Practice.

[ref-43] Zaworski K, Latosiewicz R (2021). The effectiveness of manual therapy and proprioceptive neuromuscular facilitation compared to kinesiotherapy: a four-arm randomized controlled trial. European Journal of Physical and Rehabilitation Medicine.

[ref-44] Zhang G, Huang Q, Gu R, Liu S, Hu C, Liu K (2023). Comparative effect of different neuromuscular exercises on pain and motor function of knee in patients with early knee osteoarthritis. Chinese Journal of Rehabilitation Theory and Practice.

[ref-45] Zhang K, Li Y, Zhao F, Xie X, Yan S, Kong D, Zhang W, Zhou J, Ma H (2025). The efficacy and safety of pilates exercise in patients with knee osteoarthritis: a systematic review with meta-analysis of randomized controlled trials. Annals of Medicine.

[ref-46] Zhu G-C, Chen K-M, Belcastro F (2024). Comparing different stretching exercises on pain, stiffness, and physical function disability in older adults with knee osteoarthritis. Archives of Physical Medicine and Rehabilitation.

[ref-47] Zhu S, Wang Z, Liang Q, Zhang Y, Li S, Yang L, He C, Chinese Society of Physical Medicine and Rehabilitation, West China Hospital (2023). Chinese guidelines for the rehabilitation treatment of knee osteoarthritis: an CSPMR evidence-based practice guideline. Journal of Evidence-Based Medicine.

[ref-48] Zifchock RA, Kirane Y, Hillstrom H, Hospital for Special Surgery Lower Extremity Realignment Research Group (2011). Are joint structure and function related to medial knee OA pain? A pilot study. Clinical Orthopaedics and Related Research.

