# Supplementary material for: Effect of proprioceptive neuromuscular facilitation on pain and joint mobility in knee osteoarthritis: a systematic review and meta-analysis of randomized controlled trials
Source: PeerJ. 2026 Jan 16;14:e20581. doi: 10.7717/peerj.20581 (PMC12814904; doi:10.7717/peerj.20581)
Supplement: Supplemental Information 1 [file peerj-14-20581-s001.docx]

**Supplemental Online Content**

Table S1. Search strategy

Table S2. Detailed characteristics of all included studies

Table S3. Certainty of evidence assessed by GRADE approach

Table S4. Study-level effects for PNF vs RT (no pooling)

Figure S1. Risk of bias assessment.

Figure S2. Forest plot: Comparison of VAS score changes from baseline to post-intervention between the PNF and control groups

Figure S3. Trial sequential analysis (TSA) of VAS comparing the PNF and control groups

Figure S4. Leave-one-out sensitivity analysis (PNF vs. control) for pain score

Figure S5. Forest plots comparing PNF vs. control on pain score under two combined SD coefficients

Figure S6. TSA of knee AROM comparing the PNF and control groups

Figure S7. Forest plots comparing PNF vs. control on knee AROM under two combined SD coefficients

**Table S1**

**Pubmed Search Strategy**

|  |  | 2024-4-9 | 2025-8-15 |
| --- | --- | --- | --- |
| #1 | ((knee osteoarthritis) OR (knee osteoarthritides) OR (gonarthriti*) OR (osteoarthriti*) OR (osteoarthro*) OR (gonarthro*)) | 124,406 | 138,451 |
| #2 | ((proprioceptive neuromuscular facilitation*) OR (proprioceptive neuromuscular facilitation training*) OR (Neuromuscular facilitation*) OR (neuromuscular stimulation*) OR (autegenic inhibition*) OR (reciprocal inhibition*) OR (rhytmic stabilization*) OR (repeat contraction*) OR (hold relax*) OR (antagonist contract*) OR (slow reversal*) OR (contract relax*)) | 1,128,405 | 1,201,505 |
| #3 | (randomized controlled trial[Publication Type] OR randomized[Title/Abstract] OR placebo[Title/Abstract]) | 1,044,170 | 1,141,061 |
| #4 | #1 AND #2 AND #3 | 223 | 239 |

**Embase Search Strategy**

|  |  | 2024-4-9 | 2025-8-15 |
| --- | --- | --- | --- |
| #1 | ‘knee osteoarthritis’:ab,ti OR ‘knee osteoarthritides’:ab,ti OR ‘gonarthriti*’:ab,ti OR ‘osteoarthriti*’:ab,ti OR ‘osteoarthro*’:ab,ti OR ‘gonarthro*’:ab,ti | 136,054 | 158,175 |
| #2 | 'proprioceptive neuromuscular facilitation*' OR 'proprioceptive neuromuscular facilitation training*' OR 'neuromuscular facilitation*' OR 'neuromuscular stimulation*' OR 'autegenic inhibition*' OR 'reciprocal inhibition*' OR 'rhytmic stabilization*' OR 'repeat contraction*' OR 'hold relax*' OR 'antagonist contract*' OR 'slow reversal*' OR 'contract relax*' OR 'PNF' | 5,992 | 7,013 |
| #3 | 'randomized controlled trial':ab,ti OR 'randomized':ab,ti OR 'placebo':ab,ti | 1,167,900 | 1,497,061 |
| #4 | #1 AND #2 AND #3 | 16 | 32 |

**Search Strategy of Web of Science**

|  |  | 2024-4-9 | 2025-8-15 |
| --- | --- | --- | --- |
| #1 | TS= (knee osteoarthritis OR knee osteoarthritides OR gonarthriti* OR osteoarthriti* OR osteoarthro* OR gonarthro*) | 189,806 | 208,462 |
| #2 | TS= (proprioceptive neuromuscular facilitation* OR proprioceptive neuromuscular facilitation training* OR Neuromuscular facilitation* OR neuromuscular stimulation* OR autegenic inhibition* OR rhytmic stabilization* OR reciprocal inhibition* OR repeat contraction* OR hold relax* OR antagonist contract* OR slow reversal* OR contract relax*) | 119,527 | 130,538 |
| #3 | #1 AND #2 | 461 | 495 |

**Cochrane Search Strategy**

|  |  | 2024-4-9 | 2025-8-15 |
| --- | --- | --- | --- |
| #1 | (knee osteoarthritis):ti,ab,kw OR (knee osteoarthritides):ti,ab,kw OR (gonarthriti*):ti,ab,kw OR (osteoarthriti*):ti,ab,kw OR (osteoarthro*):ti,ab,kw OR (gonarthro*):ti,ab,kw | 240,49 | 271,17 |
| #2 | (proprioceptive neuromuscular facilitation*):ti,ab,kw OR (proprioceptive neuromuscular facilitation training*):ti,ab,kw OR (Neuromuscular facilitation*):ti,ab,kw OR (neuromuscular stimulation*):ti,ab,kw OR (autegenic inhibition*):ti,ab,kw OR (rhytmic stabilization*):ti,ab,kw OR (reciprocal inhibition*):ti,ab,kw OR (repeat contraction*):ti,ab,kw OR (hold relax*):ti,ab,kw OR (antagonist contract*):ti,ab,kw OR (slow reversal*):ti,ab,kw OR (contract relax*):ti,ab,kw | 5,226 | 5,982 |
| #3 | #1 AND #2 | 167 | 194 |

**China National Knowledge Infrastructure (CNKI)**

|  | 2024-4-9 | 2025-8-15 |
| --- | --- | --- |
| SU = ('PNF' + 'Proprioceptive Neuromuscular Facilitation') AND SU = ('OA' + 'Osteoarthritis' + 'Knee Osteoarthritis' + 'Knee Osteoarthropathy' + 'Knee Arthritis' + 'Proliferative Arthritis' + 'Degenerative Osteoarthropathy' + 'Senile Arthritis' + 'Degenerative Arthritis' + 'Hypertrophic Osteoarthritis' + 'Degenerative Arthritis' + 'Degenerative Arthritis' + 'Degenerative Arthritis of the Knee') | 25 | 28 |
| Limits: Chinese literature |  |  |

**Wangfang**

|  | 2024-4-9 | 2025-8-15 |
| --- | --- | --- |
| (Topic:(PNF) or Topic:( Proprioceptive Neuromuscular Facilitation)) and (Topic:(OA) or Topic:(Osteoarthritis) or Topic:(Knee Osteoarthritis) or Topic:(Knee Osteoarthropathy) or Topic:(Knee Arthritis) or Topic:(Proliferative Arthritis) or Topic:(Proliferative Arthritis) or Topic:(Degenerative Osteoarthropathy) or Topic:(Senile Arthritis) or Topic:(Degenerative Arthritis) or Topic:(Hypertrophic Osteoarthritis) or Topic:(Degenerative Arthritis) or Topic:(Degenerative Arthritis) or Topic:(Degenerative Arthritis of the Knee)) | 18 | 34 |
| Limits: Chinese literature |  |  |

**VIP**

|  | 2024-4-9 | 2025-8-15 |
| --- | --- | --- |
| (M= PNF OR M= Proprioceptive Neuromuscular Facilitation) AND (M= OA OR M= Osteoarthritis OR M= Knee Osteoarthritis OR M= Knee Osteoarthropathy OR M= Knee Arthritis OR M= Proliferative Arthritis OR M= Proliferative Arthritis OR M= Degenerative Osteoarthropathy OR M=Senile Arthritis OR M= Degenerative Arthritis OR M= Hypertrophic Osteoarthritis OR M= Degenerative Arthritis OR M= Degenerative Arthritis OR M= Degenerative Arthritis of the Knee) | 14 | 18 |

**Search Strategy for Chinese Databases**

The search strategy was tailored to the specific syntax of each Chinese database. The following keywords and their synonyms were utilized to ensure comprehensiveness:

- PNF-related terms: PNF, Proprioceptive Neuromuscular Facilitation, 本体感觉神经肌肉促进, 本体感觉神经肌肉刺激
- Osteoarthritis (OA)-related terms: OA, Osteoarthritis, 骨关节炎(Osteoarthritis), 骨性关节炎(Osteoarthritis), 增生性骨关节炎 (Hypertrophic Osteoarthritis), 退行性骨关节病 (Degenerative Osteoarthropathy), 老年性关节炎 (Senile Arthritis), 退行性关节炎 (Degenerative Arthritis), 增生性关节炎(Proliferative Arthritis), 退变性关节炎 (Degenerative Arthritis), 退化性关节炎 (Degenerative Arthritis)
- Knee osteoarthritis (KOA)-related terms: 膝骨关节炎(Knee Osteoarthritis), 膝骨关节病(Knee Osteoarthropathy), 膝关节炎(Knee Arthritis), 膝关节骨性关节炎(Knee Osteoarthritis), 膝关节退行性关节炎(Degenerative Arthritis of the Knee)

**Rationale of the Search Strategy:**

The core objective of the search strategy was to identify all relevant studies that investigate the application of **Proprioceptive Neuromuscular Facilitation (PNF)** for managing **Knee Osteoarthritis (KOA)**. To capture all literature on the topic, the search was designed to include both general osteoarthritis (OA) concepts and knee-specific OA (KOA) concepts.

The Boolean logic was applied as follows:

1. All synonyms within the **general OA-related terms** group were combined with **OR**.
2. All synonyms within the **knee-specific OA (KOA)-related terms** group were combined with **OR**.
3. These two groups were then combined with each other using OR to create a comprehensive Osteoarthritis concept.
4. Finally, this combined Osteoarthritis concept was connected to the **PNF-related terms** group (where all synonyms were also combined with **OR**) using **AND**.

This approach ensures the retrieval of records that mention PNF in conjunction with either general OA or specifically knee-related OA, thereby maximizing the relevance and comprehensiveness of the search results.

**1. China National Knowledge Infrastructure (CNKI)**

- Field Used: SU (Subject)
- Strategy (Chinese version):
  SU=('PNF' + '本体感觉神经肌肉促进' + '本体感觉神经肌肉刺激') **AND** SU=( OA'+'骨关节炎'+'骨性关节炎'+'膝骨关节炎'+'膝骨关节病'+'膝关节炎'+'膝关节骨性关节炎'+'增生性骨关节炎'+'退行性骨关节病'+'老年性关节炎'+'退行性关节炎'+'增生性关节炎'+'退变性关节炎'+'退化性关节炎'+'膝关节退行性关节炎')
- Strategy (English version):

SU= ('PNF' + 'Proprioceptive Neuromuscular Facilitation') **AND** SU= ('OA' + 'Osteoarthritis' + 'Knee Osteoarthritis' + 'Knee Osteoarthropathy' + 'Knee Arthritis' + 'Proliferative Arthritis' + 'Degenerative Osteoarthropathy' + 'Senile Arthritis' + 'Degenerative Arthritis' + 'Hypertrophic Osteoarthritis' + 'Degenerative Arthritis' + 'Degenerative Arthritis' + 'Degenerative Arthritis of the Knee')

- Limits: Chinese literature.

**2. Wanfang Data (Wanfang)**

- Field Used: Topic (主题)
- Strategy (Chinese version):
  (主题:(OA) or 主题:(骨关节炎) or 主题:(骨性关节炎) or 主题:(膝骨关节炎) or 主题:(膝骨关节病) or 主题:(膝关节炎) or 主题:(膝关节骨性关节炎) or 主题:(增生性骨关节炎) or 主题:(退行性骨关节病) or 主题:(老年性关节炎) or 主题:(退行性关节炎) or 主题:(增生性关节炎) or 主题:(退变性关节炎) or 主题:(退化性关节炎) or 主题:(膝关节退行性关节炎)) and (主题:(PNF) or 主题:(本体感觉神经肌肉促进) or 主题:(本体感觉神经肌肉刺激))
- Strategy (English version):

(Topic:(PNF) or Topic:(Proprioceptive Neuromuscular Facilitation)) **and** (Topic:(OA) or Topic:(Osteoarthritis) or Topic:(Knee Osteoarthritis) or Topic:(Knee Osteoarthropathy) or Topic:(Knee Arthritis) or Topic:(Proliferative Arthritis) or Topic:(Proliferative Arthritis) or Topic:(Degenerative Osteoarthropathy) or Topic:(Senile Arthritis) or Topic:(Degenerative Arthritis) or Topic:(Hypertrophic Osteoarthritis) or Topic:(Degenerative Arthritis) or Topic:(Degenerative Arthritis) or Topic:(Degenerative Arthritis of the Knee))

- Limits: Journal articles.

**3. VIP Data (VIP)**

- Field Used: M (Subject/主题)
- Strategy(Chinese version):
  (M=PNF OR M=本体感觉神经肌肉刺激 OR M=本体感觉神经肌肉促进) **AND** (M=膝关节骨性关节炎 OR M=膝骨关节炎 OR M=骨关节炎 OR M=增生性骨关节炎 OR M=膝关节骨关节 OR M=退行性骨关节病 OR M=骨关节病 OR M=退行性关节病 OR M=老年性关节炎 OR M=退行性关节炎 OR M=骨性关节病 OR M=增生性关节炎 OR M=退变性关节炎 OR M=退化性关节炎 OR M=膝关节退行性关节炎 OR M=骨性关节炎 OR M=膝关节骨关节炎 OR M=膝骨关节病)
- Strategy(English version):
  (M= PNF OR M= Proprioceptive Neuromuscular Facilitation) **AND** (M= OA OR M= Osteoarthritis OR M= Knee Osteoarthritis OR M= Knee Osteoarthropathy OR M= Knee Arthritis OR M= Proliferative Arthritis OR M= Proliferative Arthritis OR M= Degenerative Osteoarthropathy OR M=Senile Arthritis OR M= Degenerative Arthritis OR M= Hypertrophic Osteoarthritis OR M= Degenerative Arthritis OR M= Degenerative Arthritis OR M= Degenerative Arthritis of the Knee)

**Table S2 Detailed characteristics of all included studies**

| **Study** | **Country** | **N (I/C)** | **Characteristics of I/C** | **I/C details** | **Intervention schedule** | **Outcomes** |
| --- | --- | --- | --- | --- | --- | --- |
| **Trials comparing PNF and control interventions** | | | |  |  |  |
| Shen 2024 | China | 27 (14/13) | Age (I/C): 65.3±4.6/66.6±7.0 Average age: 65.9±5.8 Gender (I/C): M:10/9; F:4/4 K/L grade: Ⅰ,Ⅱ,Ⅲ Affected limb: unilateral or bilateral | Intervention: PNF stretching performed using specialized PNF techniques, including HR, CR, DR and RC, within four lower limb spiral-diagonal movement patterns Comparator: health education | Duration: 6 wk Time: 60 min Frequency: 3/wk | VAS,  knee AROM |
| Gao 2023 | China | 27 (13/14) | Age (I/C): 68.5±2.1/67.9±1.4 Average age: 68.2±1.8 Gender (I/C): M:8/8; F:5/6 K/L grade: Ⅱ,Ⅲ,Ⅳ Affected limb: unilateral or bilateral | Intervention: PNF stretching performed using specialized PNF techniques, including HR, CR, DR and RC, within four lower limb spiral-diagonal movement patterns Comparator: health education | Duration: 8 wk Time: 60 min Frequency: 3/wk | VAS |
| Song 2020 | China | 29 (13/16) | Age (I/C): 68.5±4.3/67.4±3.4 Average age: 67.9±3.8 Gender (I/C): M:5/6; F:8/10 K/L grade: Ⅰ,Ⅱ,Ⅲ | Intervention: PNF stretching performed using specialized PNF techniques, including HR, CR, DR and RC, within four lower limb spiral-diagonal movement patterns Comparator: television viewing or magazines reading | Duration: 12 wk Time: 60 min Frequency: 3/wk | VAS-WOMAC |
| Li 2018 | China | 60(30/30) | Age: 63.5±8.8/64.3±9.2  Average age:63.9±8.9  Gender: M:10/11; F:20/19  K/L grade: Ⅱ, Ⅲ, Ⅳ | Intervention: specialized PNF techniques, including HR, CR, DR and RS  Comparator: no intervention  Baseline intervention: interferential therapy and magnetic field-vibration heating  Affected limb: unilateral | Duration: 2 wk  Time: 30 min  Frequency: 5/wk | VAS |
| Weng 2009 | China | 58 (30/28) | K/L grade: Ⅱ,Ⅲ^#^ Affected limb: bilateral | Intervention: specialized PNF techniques for the quadriceps and the biceps femoris, including HR and CR Comparator: no intervention Baseline intervention: isokinetic exercise, hot packs and passive ROM exercise | Duration: 8 wk Time: 10 min Frequency: 3/wk | VAS,  knee AROM |
| **Trials comparing PNF and rehabilitation technique (RT)** | | | | |  |  |
| Anjum 2023 | Pakistan | 57 (27/30) | Age (I/C): 44.3±4.9/45.9±4.6 Average age: 45.1±4.8 Gender (I/C): M:9/5; F:18/25 K/L grade: Ⅰ,Ⅱ | Intervention: specialized PNF techniques for the hamstring muscle, including HR, CR and RC Comparator: instrument assisted soft tissue mobilization Baseline intervention: isometric quadriceps exercises | Duration: 6 wk Time: 30 min Frequency: 3/wk | VAS |
| Zhang 2023 | China | 40(20/20) | Age (I/C): 56.6±4.0/57.9±4.1  Average age: 57.3±4.1  Gender (I/C): M:8/8; F:12/12  K/L grade: Ⅰ, Ⅱ | Intervention: PNF stretching performed using specialized PNF techniques, including HR, CR, DR, RS, RI, SR and COI, within four lower limb spiral-diagonal movement patterns  Comparator: neuromuscular exercise, including exercises such as glute bridge, step-ups, weight shifting, squats, side plank, standing resistance, lunges, single-leg squats, and kettlebell swings | Duration: 6 wk  Time: 60 min  Frequency: 2/wk | VAS, knee AROM |
| Nafees 2023 | Pakistan | 48 (24/24) | Age (I/C): 56.8±8.8/52.1±7.1 Average age: 54.5±8.3 Gender (I/C): M:8/3; F:16/21 Affected limb: bilateral | Intervention: PNF stretching performed using specialized PNF techniques, including HR Comparator: dynamic assisted soft tissue mobilization Baseline intervention: cryo therapy, isometric quadriceps and isometric hip adductor strengthening exercises | Duration: 4 wk Time: 30 min Frequency: 3/wk | VAS |
| Weng 2009 | China | 57 (30/27) | K/L grade: Ⅱ,Ⅲ^#^ Affected limb: bilateral | Intervention: specialized PNF techniques for the quadriceps and the biceps femoris, including HR and CR Comparator: bilateral knee static stretching for the affected knee Baseline intervention: isokinetic exercise after hot-packing the knee | Duration: 8 wk Time: 10 min Frequency: 3/wk | VAS,  knee AROM |
| Abbreviations: I/C, intervention/comparator; K/L Grade, Kellgren–Lawrence grade; TKA, total knee arthroplasty; PNF, proprioceptive neuromuscular facilitation; HR, hold relax; CR, contraction relax; DR, dynamic reversal; RC, repeated contraction; RS, rhythmic stabilization; RI, rhythmic initiation; SR, stability reversal; COI, combination of isotonics; wk, week; VAS, visual analogue scale; AROM, active range of motion used when the knee flexes ; WOMAC, Western Ontario and McMaster Universities Osteoarthritis Index; NPRS, numerical pain rating scale. Quantitative data are presented as mean ± SD. | | | | | | |
| ^#^The K/L grade was derived from the equivalent Altman Grade II classification. | | | | | | |

**Figure S1 Risk of bias assessment** **using the RoB 2 tool**

Abbreviations: PNF, Proprioceptive Neuromuscular Facilitation; RT, rehabilitation technique; VAS, Visual Analogue Scale; AROM, active range of motion; NPRS, Numerical Pain Rating Scale.

**
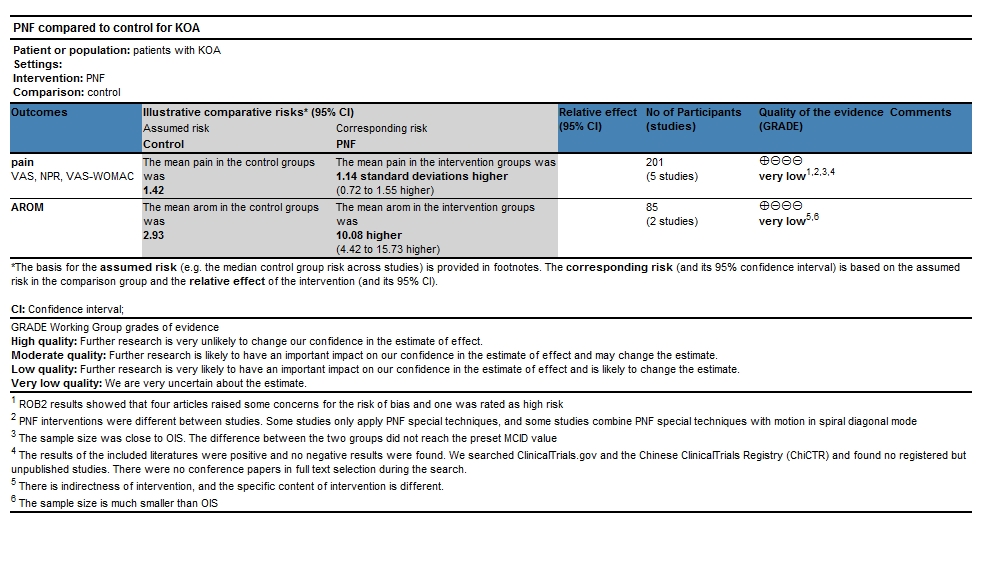
Table S3 Certainty of evidence assessed by GRADE approach**

**Table S4 Study-level effects for PNF vs RT (no pooling)**

| **Study** | **RT type** | **Outcome** | **Mean±SD of change**  **（PNF）** | **Mean±SD of change**  **(RT)** | **MD of change (95% CI)** |
| --- | --- | --- | --- | --- | --- |
| Anjum 2023 | soft tissue mobilization | VAS | -3.61±1.20 | -6.20±1.10 | 2.59 (1.99, 3.19) |
| Zhang 2023 | neuromuscular training | VAS | -3.19±1.03 | -0.98±1.21 | -2.21 (-2.91, -1.51) |
| Nafees 2023 | soft tissue mobilization | VAS | -5.31±1.27 | -5.11±1.36 | -0.2 (-0.95, 0.545) |
| Weng 2009 | combined quadriceps and hamstring stretching | VAS | -2.20±1.71 | -1.60±1.06 | -0.6 (-1.33, 0.13) |
| Zhang 2023 | neuromuscular training | knee AROM | 7.13±5.69 | 3.26±5.65 | 3.87 (0.36, 7.38) |
| Weng 2009 | combined quadriceps and hamstring stretching | knee AROM | 17.0±16.50 | 10.0±14.40 | 7 (-1.03, 15.03) |

* r=0.5 for change SD

**Figure S2 Forest plot: Comparison of VAS score changes from baseline to post-intervention between the PNF and control groups**

**
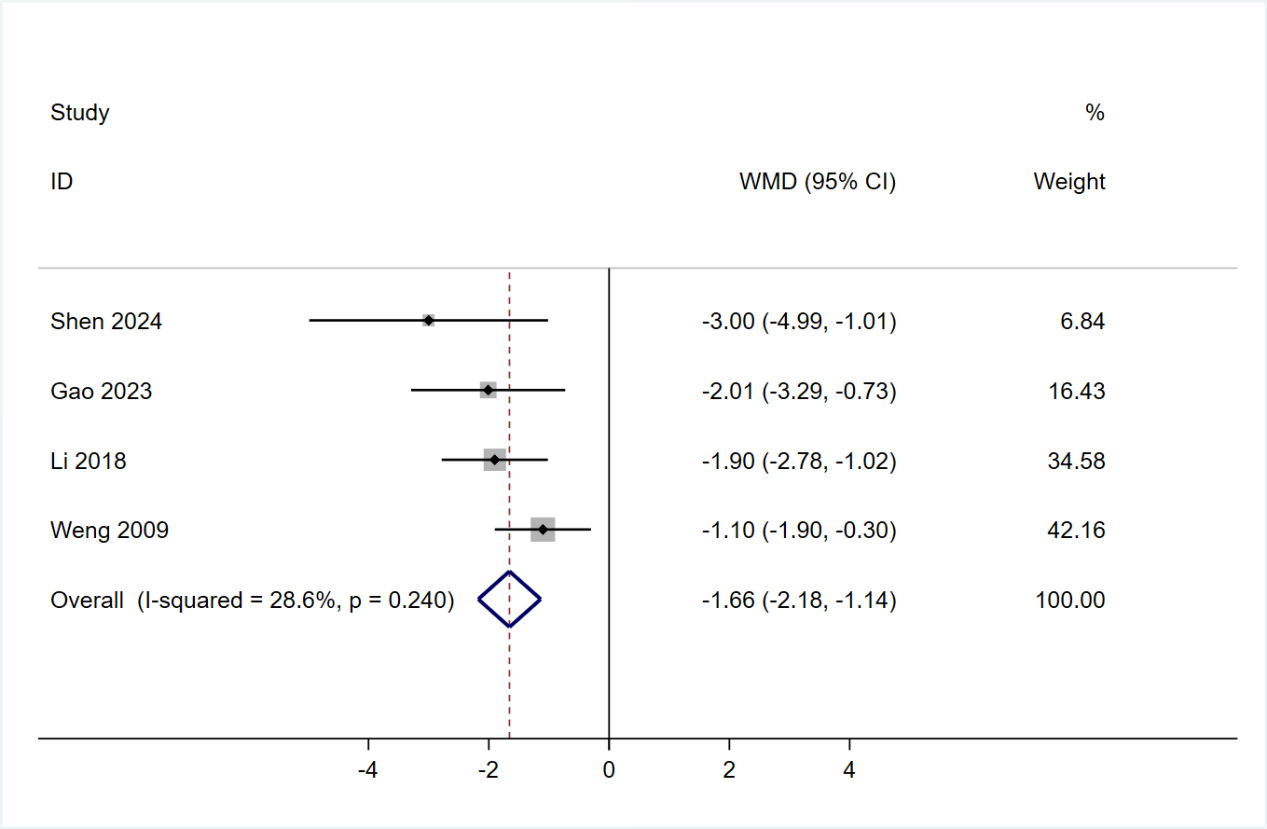
**

**
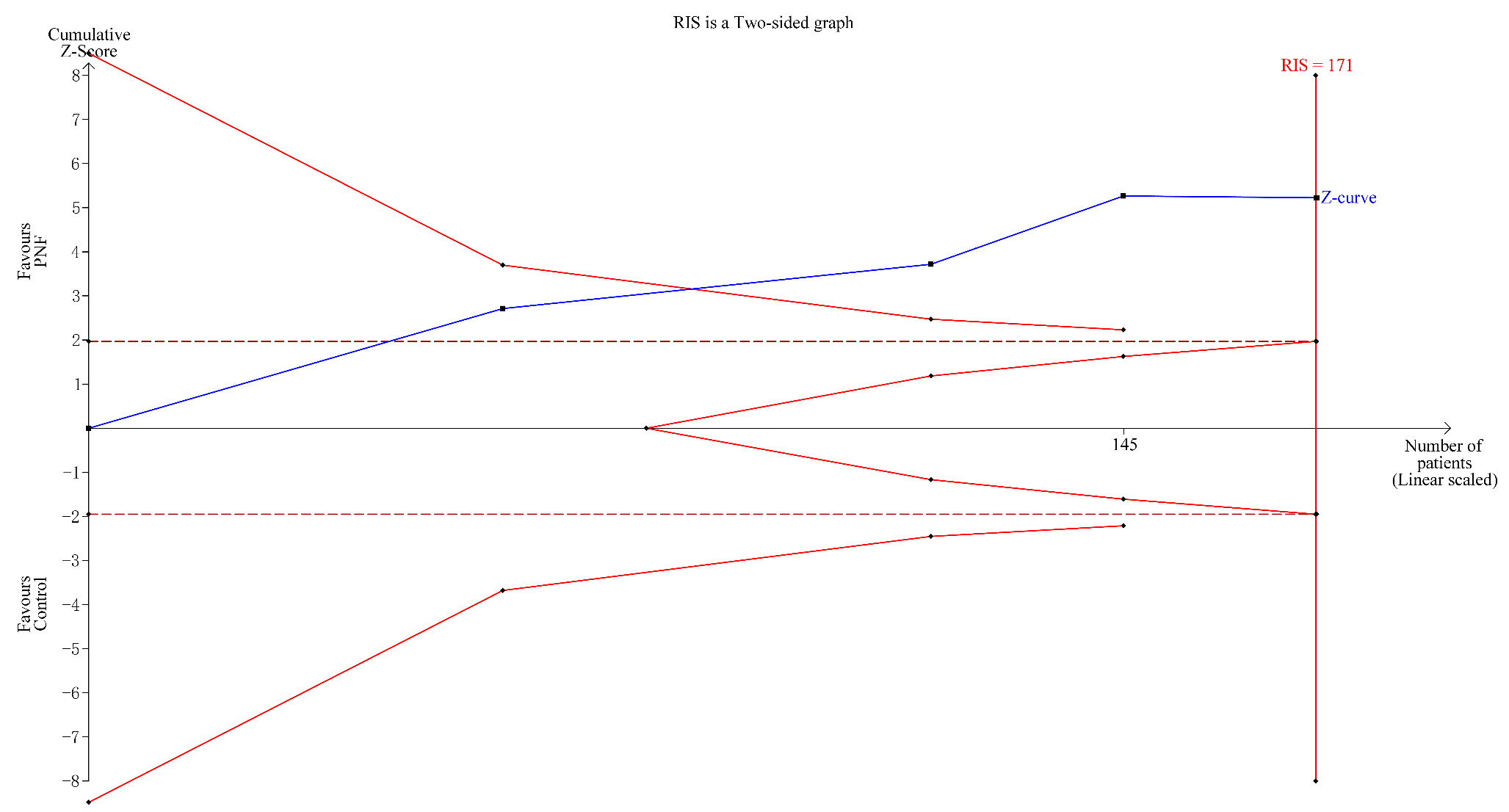
Figure S3 Trial sequential analysis (TSA) of VAS comparing the PNF and control groups**

Trial sequential analysis was performed on the original studies reporting VAS outcomes (excluding Alaca 2015), comparing PNF with the control group. The heterogeneity-adjusted required information size (RIS) was 171 participants, calculated based on the empirical variance, a mean difference of –1.14, α = 0.05, β = 0.10, and I² = 43%. The blue cumulative z-curve was constructed using a DerSimonian–Laird random-effects model and crossed both the RIS boundary and the conventional monitoring boundaries.

**
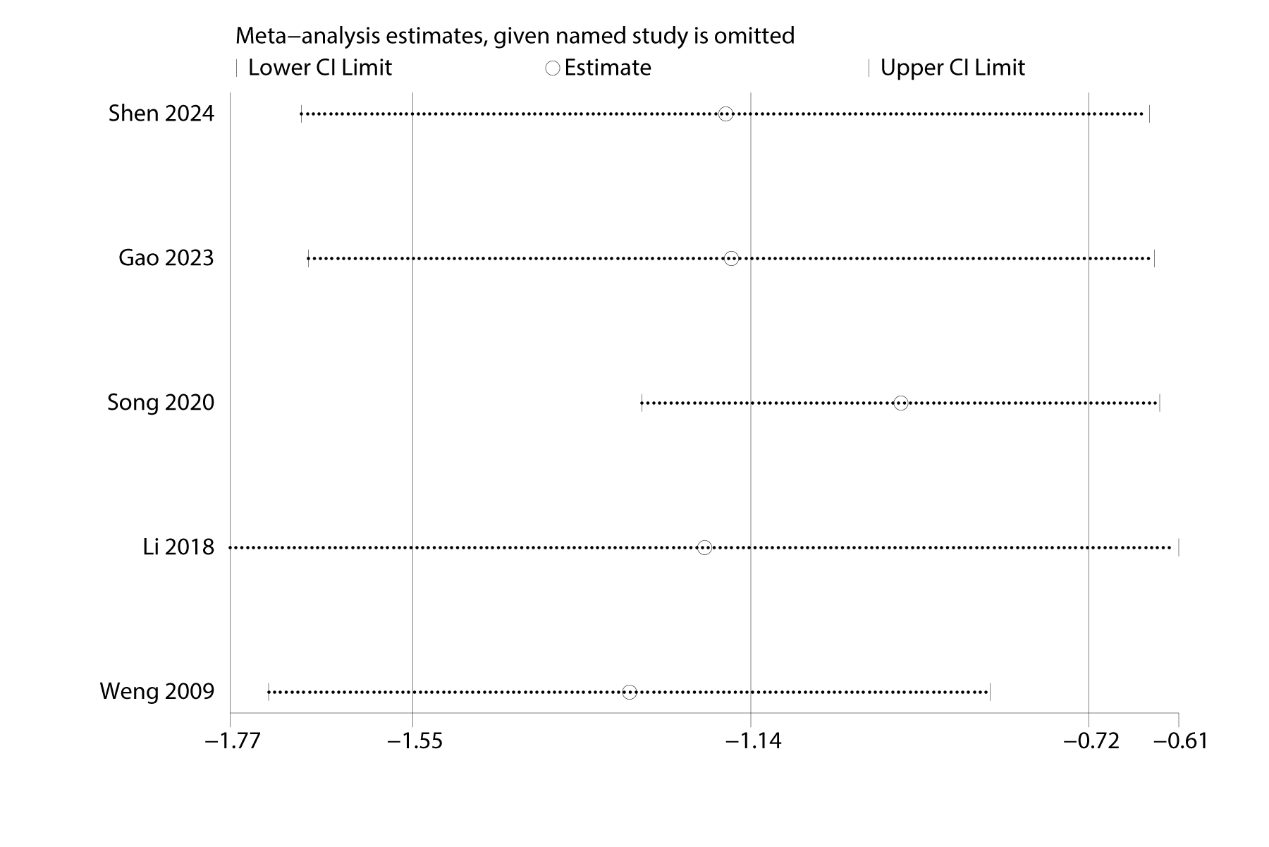
Figure S4 Leave-one-out sensitivity analysis (PNF vs. control) for pain score**

**Figure S5 Forest plots comparing PNF vs. control on pain score under two combined SD coefficients**

**
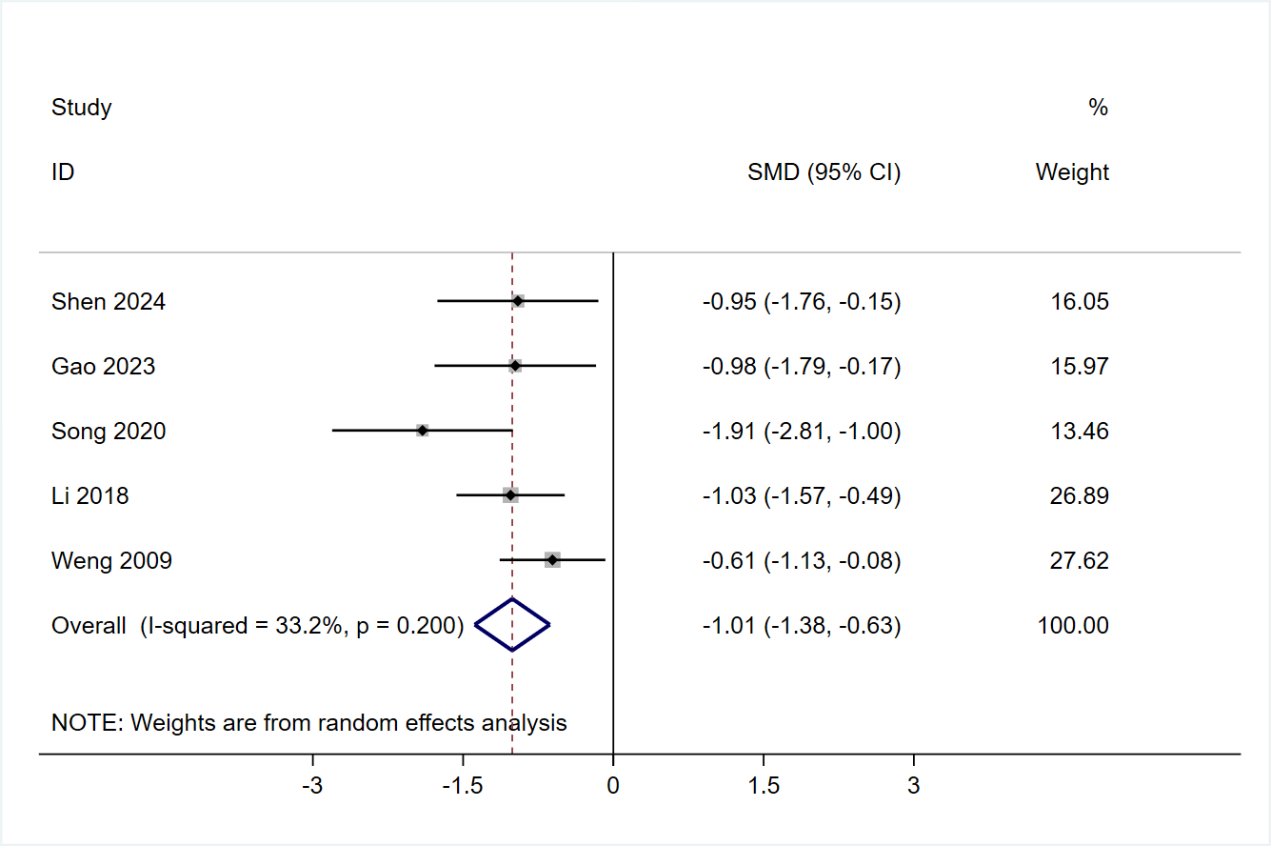

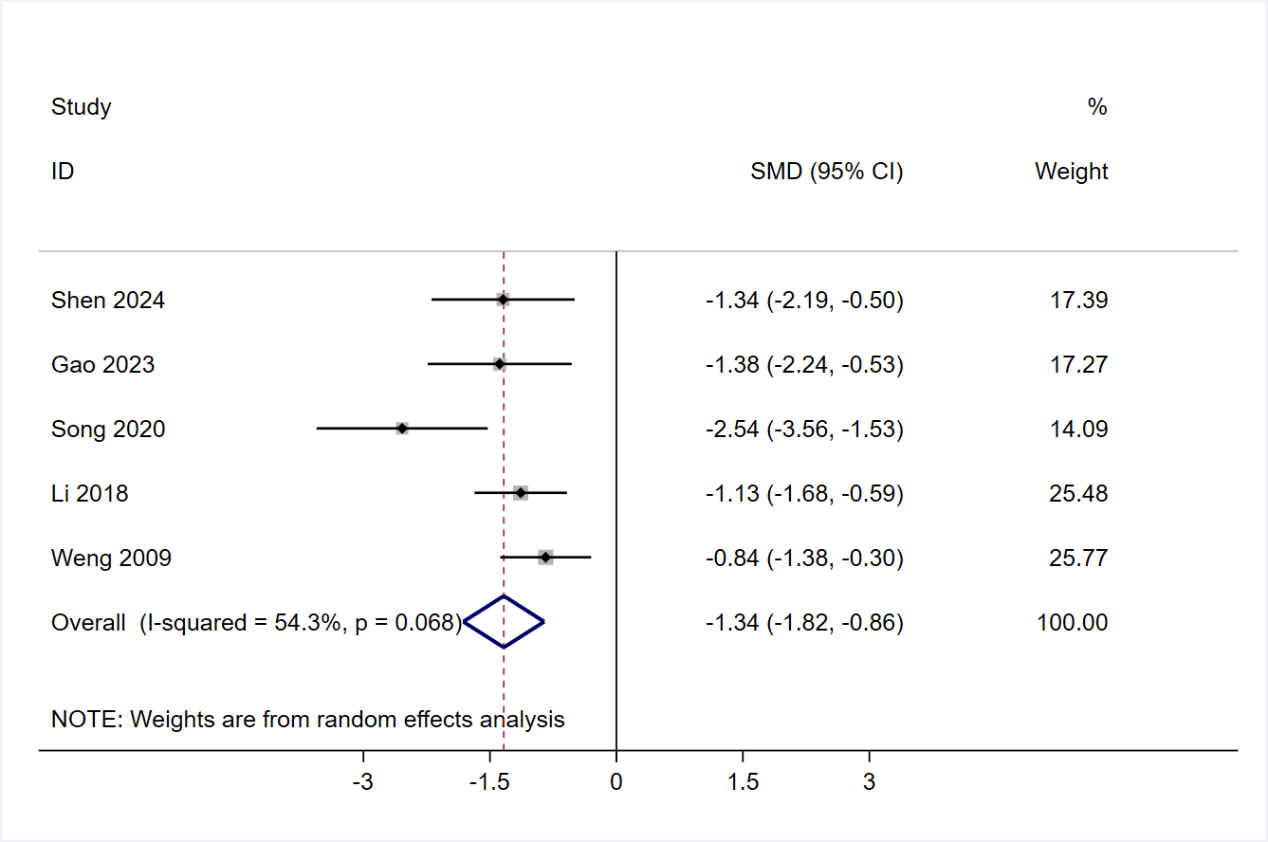
**

**B**

**A**

(A) r = 0.3 and (B) r = 0.7. Each plot displays the pooled effect size and 95% confidence interval under the respective assumption.

**
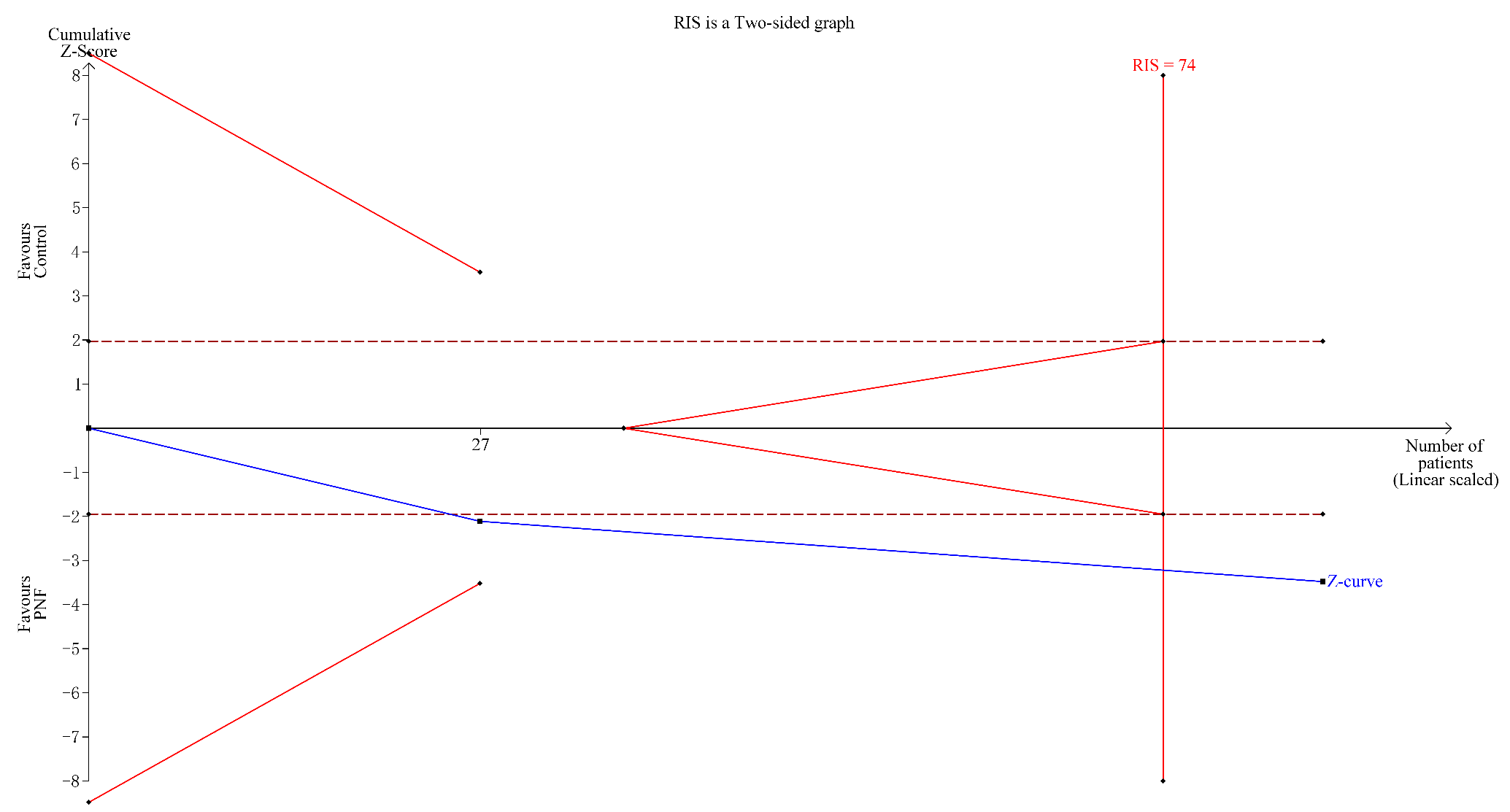
Figure S6 TSA of knee AROM comparing the PNF and control groups**

Trial sequential analysis was performed on the original studies reporting knee AROM outcomes, comparing PNF with the control group. The RIS was 74 participants, determined using the empirical variance, a mean difference of 10.08, α = 0.05, β = 0.10, and a model variance-based heterogeneity estimate. The blue cumulative z-curve was constructed using a DerSimonian–Laird random-effects model and crossed both the RIS boundary and the conventional monitoring boundaries.

**Figure S7 Forest plots comparing PNF vs. control on knee AROM under two combined SD coefficients**

**
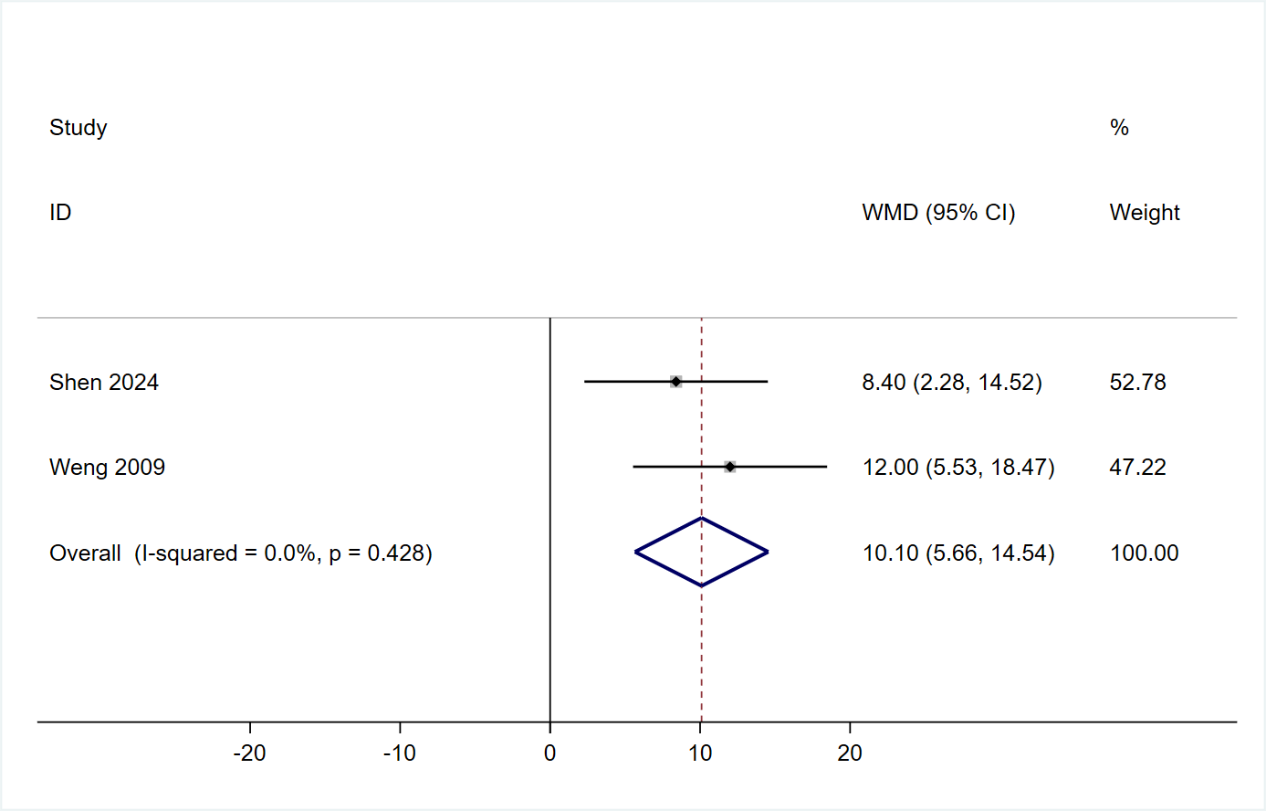

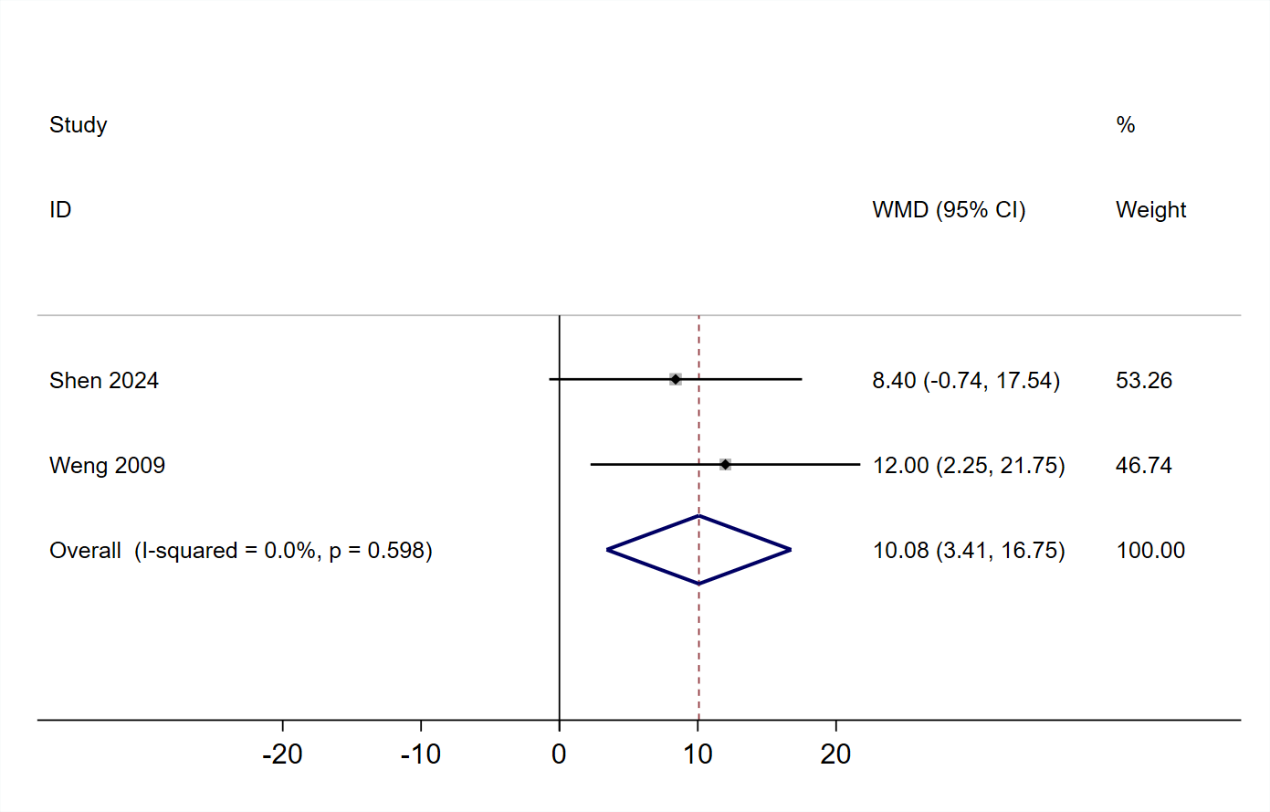
**

**B**

**A**

(A) r = 0.3 and (B) r = 0.7. Each plot displays the pooled effect size and 95% confidence interval under the respective assumption.
