# Supplementary material for: Effect of proprioceptive neuromuscular facilitation on pain and joint mobility in knee osteoarthritis: a systematic review and meta-analysis of randomized controlled trials
Source: PeerJ. 2026 Jan 16;14:e20581. doi: 10.7717/peerj.20581 (PMC12814904; doi:10.7717/peerj.20581)
Supplement: Supplemental Information 6 [file peerj-14-20581-s006.docx]

Systematic Review and/or Meta-Analysis Rationale

Intended Audience:

The target audience for the article *"Effect of proprioceptive neuromuscular facilitation on pain and joint mobility in knee osteoarthritis: a systematic review and meta-analysis of randomized controlled trials"* would likely include:

1. **Physical Therapists and Rehabilitation Specialists:** Professionals interested in evidence-based strategies to enhance patient outcomes related to joint mobility and pain management in knee osteoarthritis through proprioceptive neuromuscular facilitation (PNF).
2. **Clinicians in Multidisciplinary Pain Management:** Healthcare providers such as physiatrists and pain specialists who seek to expand non-pharmacological treatment options for knee osteoarthritis, particularly when pharmacological approaches are ineffective or accompanied by significant side effects.
3. **Sports Medicine Practitioners:** Clinicians and trainers working with athletes or physically active individuals aiming to integrate preventive and therapeutic rehabilitation techniques, including PNF, to mitigate or manage knee osteoarthritis.
4. **Orthopedic Surgeons**: Specialists in joint surgery and musculoskeletal care who can utilize the findings to inform rehabilitation protocols both pre- and post-operatively, optimizing recovery outcomes for patients with knee osteoarthritis.
